# Optimization of an ammonia assay based on transmembrane pH-gradient polymersomes

**DOI:** 10.1038/s41598-021-01137-1

**Published:** 2021-11-11

**Authors:** Anastasia Spyrogianni, Charlotte Gourmel, Leopold Hofmann, Jessica Marbach, Jean-Christophe Leroux

**Affiliations:** Department of Chemistry and Applied Biosciences, Institute of Pharmaceutical Sciences, ETH Zurich, Vladimir-Prelog-Weg 3, 8093 Zurich, Switzerland

**Keywords:** Diagnostic markers, Prognostic markers, Encephalopathy, Liver diseases, Metabolic disorders, Polymers, Bioanalytical chemistry, Fluorescent probes, Medical and clinical diagnostics

## Abstract

Reliable ammonia quantification assays are essential for monitoring ammonemia in patients with liver diseases. In this study, we describe the development process of a microplate-based assay for accurate, precise, and robust ammonia quantification in biological fluids, following regulatory guidelines on bioanalytical method validation. The assay is based on transmembrane pH-gradient polymersomes that encapsulate a pH-sensitive ratiometric fluorophore, the fluorescence signal of which correlates with the ammonia concentration in the sample. Using a four-parameter logistic regression, the assay had a large quantification range (30–800 μM ammonia). As for selectivity, the presence of amino acids or pyruvate (up to clinically relevant concentrations) showed no assay interference. In samples with low bilirubin levels, polymersomes containing the fluorophore pyranine provided accurate ammonia quantification. In samples with high bilirubin concentrations, billirubin’s optical interference was alleviated when replacing pyranine with a close to near-infrared hemicyanine fluorophore. Finally, the assay could correctly retrieve the ammonia concentration in ammonia-spiked human plasma samples, which was confirmed by comparing our measurements with the data obtained using a commercially available point-of-care device for ammonia.

## Introduction

Blood ammonia is increasingly considered a central prognostic analyte in a number of life-threatening conditions^1–3^. Ammonia is a potentially neurotoxic compound that is mainly produced by the catabolism of amino acids and the hydrolysis of urea in the gastrointestinal tract^4^. In healthy subjects, blood ammonia is kept at homeostasis through its involvement in the synthesis of nitrogen-containing compounds and its elimination by the urea cycle in the liver^4^. Defects in these pathways, due to inborn errors of metabolism (e.g. urea cycle disorders) or acquired diseases (e.g. liver diseases, drug toxicity), lead to an elevated ammonia level in systemic circulation^3–5^. A condition with plasma ammonia levels > 50 µM in adults and > 100 µM in newborns is defined as hyperammonemia^5,6^. Concentrations greater than 500 μM have been reported for children with inborn errors of metabolism^7^.

Patients with hyperammonemia may develop hepatic encephalopathy, which manifests as a wide spectrum of neurological or psychiatric abnormalities ranging from mild cognitive and behavioral alterations to coma^8^. Even the mildest forms of hepatic encephalopathy negatively impact the quality of life of both patients and caregivers, while overt and recurrent episodes of hepatic encephalopathy are related to poor prognosis, mortality, and increased healthcare burden^9^.

Measuring blood ammonia levels is of key importance for diagnosing inborn errors of metabolism or drug toxicity in patients presenting neurological abnormalities without apparent liver problems, and may have a prognostic value in patients with acute liver failure^1,3,10^. Despite the wide interest in developing ammonia sensors^11–14^, the manufacturing complexity or the lack of a full bioanalytical method validation hamper many—otherwise promising—technologies to meet clinical requirements and make it to the market. In clinical settings, ammonia is commonly measured in blood plasma samples using an enzymatic assay, performed in clinical chemistry analyzers^15,16^. Although many of the currently available enzymatic assays have a wide quantification range (e.g. 8.8–1174.2 μM for the L3K^®^ assay from Sekisui Diagnostics), the assay procedure is often quite complex resulting in long turnaround times^16^. Furthermore, high plasma ammonia values may be obtained as an artefact due to incorrect blood sampling and processing^7^. An alternative in vitro diagnostic method, which has been implemented both in clinical chemistry analyzers (for plasma) and as a point-of-care (POC) device (for blood), is based on sample alkalization, ammonia gas microdiffusion, and colorimetry^15,16^. While data turnaround times for POC devices are faster^16^, the quantification range is relatively narrow (7–286 μM).

**Figure 1:**
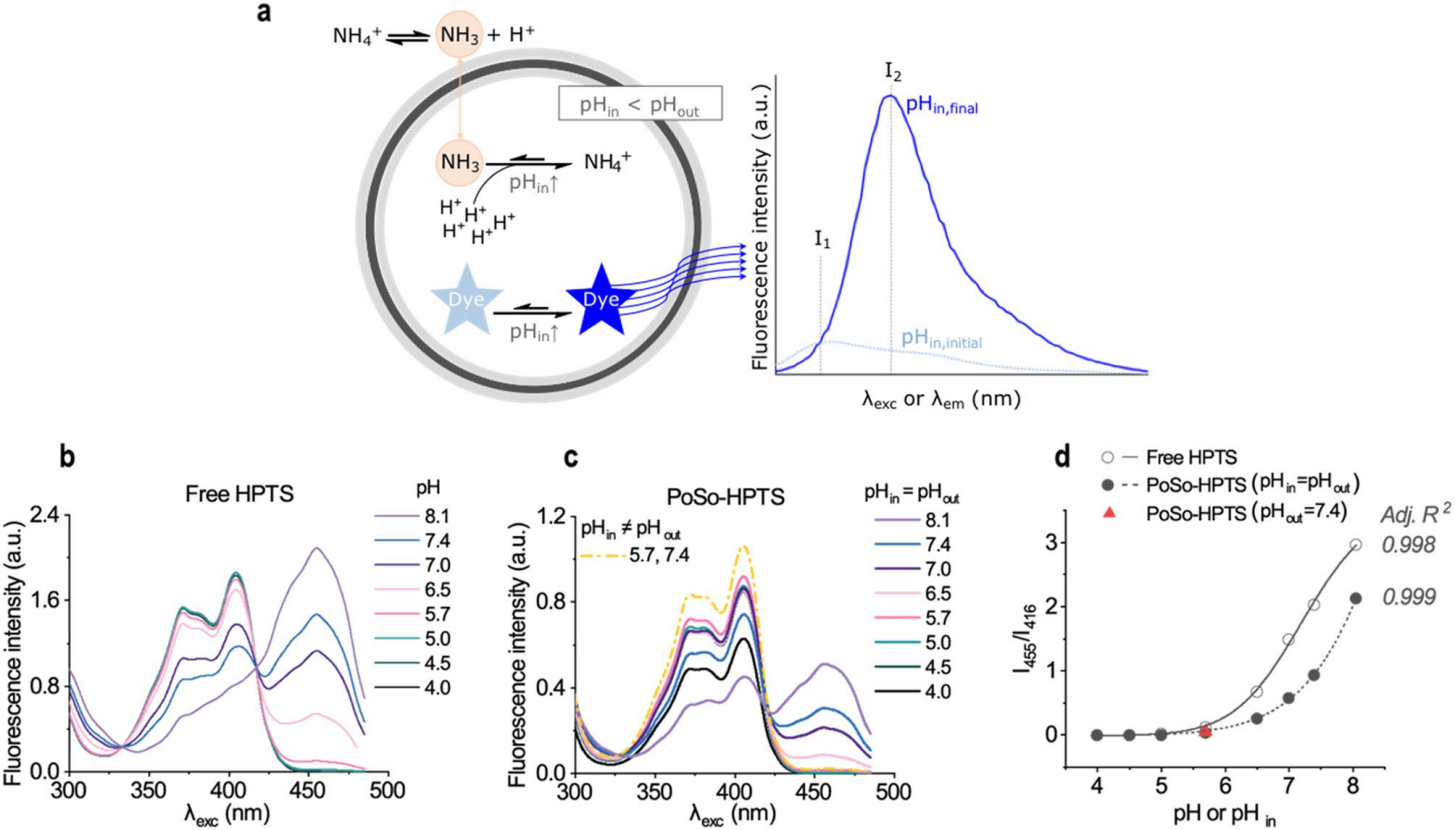
Working principle of PoSo-based ammonia assay, and fluorescence properties of free HPTS vs. PoSo-HPTS. (**a**) Once the ammonia-containing sample is mixed with the PoSo suspension, uncharged ammonia molecules diffuse into the vesicle’s acidic core, where they ionize to ammonium and then become trapped in the PoSo. The pH_in_ hence increases and the fluorescence signal of the encapsulated pH-sensitive, ratiometric dye changes. (**b**–**c**) Fluorescence excitation spectra (for λ_em_ 515 nm) of free HPTS (**b**) and PoSo-HPTS (**c**) with pH_in_ = pH_out_ (solid lines) and one sample with pH_in_ ≠ pH_out_ (dash-dotted line) for comparison. (**d**) Fluorescence intensity ratio I_455_/I_416_ at λ_em_ 515 nm (i.e. for λ_exc_ 455 and 416 nm) for free HPTS and PoSo-HPTS (symbols) against pH or pH_in_, respectively. Lines correspond to four-parameter logistic regressions.

Here, we describe the optimization process of a microplate-based assay for accurate, precise, and robust ammonia quantification in biological fluids, using transmembrane pH-gradient polymeric vesicles (polymersomes, PoSo) that encapsulate a pH-sensitive ratiometric fluorophore. PoSo were selected as they have relatively stable membranes^17^ and are less permeable and leaky^18–20^, and therefore less prone to interferences and/or loss of pH gradient than liposomes^21,22^. Once the ammonia-containing sample (e.g. plasma) is mixed with the PoSo suspension, uncharged ammonia molecules diffuse into the vesicle’s acidic core, where they ionize to ammonium and then become trapped in the PoSo^14^. The pH inside the PoSo hence increases and the fluorescence signal of the encapsulated pH-sensitive dye changes in relation to the ammonia concentration in the sample^14^ (Fig. 1a). In this study, we optimized the assay with a high standard of practice and followed regulatory guidelines on bioanalytical method validation^23–25^. Furthermore, the assay was challenged against various concentrations of potential interferents, such as amino acids^26^, pyruvate^27^, and bilirubin^28^, while its performance in human plasma was compared to a commercial POC device.

## Materials and methods

### Diblock copolymer synthesis

A poly(styrene)–poly(ethylene glycol) diblock copolymer (PS-*b*-PEG) consisting of a PEG block with a number-average molecular weight (M_n_) of 2000 Da and a poly(styrene) (PS) block with a M_n_ of 3500–4500 Da was synthesized by atom transfer radical polymerization^29,30^. Details on the synthesis are provided in the Supplementary Methods.

### Diblock copolymer characterization

The PS-*b*-PEG composition was analyzed by ^1^H NMR spectroscopy. All NMR spectra were recorded on a Bruker AV-400 spectrometer at 400 MHz (Bruker, MA, USA). Resonances are reported as chemical shifts (δ) in parts per million (ppm), and the residual undeuterated solvent peak or tetramethylsilane were used for spectral calibration. To determine the PS-*b*-PEG composition, the large peak of PEG (45 × CH_2_–CH_2_, m, δ 3.4–3.5 ppm) was compared with the aromatic styrene protons peak (n × C_6_H_5_, m, 6.2–7.2 ppm). The copolymer dispersity (Đ) was assessed by gel permeation chromatography, as detailed in the Supplementary Methods.

### PoSo preparation

A solvent emulsification method^31^ was used to prepare the PoSo. A solution of the polymer in an organic solvent [300 mg/mL PS_4k_-*b*-PEG_2k_ in dichloromethane (DCM; Acros Organics, Thermo Fisher Scientific, MA, USA)] was injected slowly into a vigorously stirred aqueous solution to form an emulsion. For transmembrane pH-gradient PoSo (made for ammonia sensing), the aqueous phase (constitutive of the PoSo inner core) consisted of citric acid (CA) buffer (5.5 mM; osmolality adjusted to 300 mOsm/kg using NaCl; pH appropriately adjusted with HCl and/or NaOH) and a pH-sensitive fluorescent dye. For PoSo made with the same inner and outer pH (pH_in_ = pH_out_; made for optical properties studies), the aqueous dye solution was prepared in CA buffer (5.5 mM) for pH_in_ ≤ 6.5, phosphate buffer (50 mM) for 6.5 < pH_in_ ≤ 8.0, and borate buffer (50 mM) for pH_in_ > 8.0. For buffer preparation, the following salts were used: CA monohydrate for analysis Emsure^®^, ACS, ISO, Reag. Ph Eur (Merck, Switzerland) for CA buffers, potassium dihydrogen phosphate for analysis Emsure^®^, ISO (Merck) for phosphate buffers and borax (Hänseler AG, Switzerland) for borate buffers.

Four different pH-sensitive fluorescent dyes were tested for encapsulation in PoSo: pyranine (8-hydroxypyrene-1,3,6-trisulfonic acid trisodium salt, HPTS), a hemicyanine dye (6-hydroxy-4-[(E)-2-(1,3,3-trimethyl-3H-1λ^5^-indol-2-yl)ethenyl]-2,3-dihydro-1H-xanthene-5,7-disulfonic acid) that was reported by Yuan et al*.*^32^ and is herein abbreviated as hCy, another hemicyanine dye that was developed as a lysosome-targeting ratiometric pH probe and referred to as Lyso-pH by Wan et al*.*^33^, and seminaphtharhodafluor (SNARF™-4F). The dye aqueous phase (1 or 3 mL) was added to a 5 mL Eppendorf tube and agitation was initiated at 30 krpm using a mechanical homogenizer (Polytron PT 1300 D, Kinematica AG, Switzerland), equipped with a PT-DA 05/2EC-E085 or PT-DA 1607/2EC probe for 1 or 3 mL batches, respectively. An appropriate volume of the polymer solution was then injected at 6 mL/h with a syringe pump (Fusion 101, Chemyx Inc, TX, USA) through a Hamilton needle (0.8 mm diameter, 80 mm length) that was bended at a 90° angle and inserted with its tip approximately in the middle of the solution. The injected polymer solution volume was 20 μL for the 1 mL PoSo-HPTS batches prepared initially (i.e. before adjusting the conditions for minimization of bilirubin interference) as well as for PoSo-Lyso and PoSo-SNARF batches. For PoSo-HPTS prepared thereafter as well as for PoSo-hCy batches, the injected polymer solution volume was 30 µL for 1 mL batches or 90 µL for 3 mL batches. After complete injection of the polymer solution, emulsification was continued for 4 or 6 min depending on batch size (1 or 3 mL, respectively). Emulsification was followed by rotary evaporation (40 °C, 700 hPa) of residual DCM for 5 or 12 min, respectively. Multiple 1 or 3 mL batches were typically prepared and pooled to obtain the desired final volume of suspension for further purification.

#### HPTS encapsulating PoSo (PoSo-HPTS)

HPTS (Acros Organics, Thermo Fisher Scientific) at concentrations ([HPTS]_in_) of 1 or 2.5 mM was used as the inner core solution. PoSo-HPTS were made for optical properties studies with pH_in_ = pH_out_ or for ammonia sensing with pH_out_ > pH_in_.

#### hCy encapsulating PoSo (PoSo-hCy)

hCy at concentrations ([hCy]_in_) of 500 or 250 μM was used as the inner core solution. The dye hCy was synthesized by Synhet Inc. (Lithuania), following a previously published method^32^. PoSo-hCy were made for optical properties studies with pH_in_ = pH_out_ or for ammonia sensing with pH_out_ > pH_in_.

#### Lyso-pH encapsulating PoSo (PoSo-Lyso)

Lyso-pH at concentrations ([Lyso-pH]_in_) of 50 or 200 μM was used as the inner core solution. The dye Lyso-pH^33^ was purchased from 007 Chemicals (The Netherlands). In PoSo-Lyso batches made with the surfactant sodium dodecyl sulfate (SDS, ACS reagent, ≥ 99.0%, Sigma-Aldrich, Merck, Switzerland), 0.09 mM SDS was added to the inner core solution. PoSo-Lyso were made for optical properties studies with pH_in_ = pH_out_.

#### SNARF™-4F encapsulating PoSo (PoSo-SNARF)

SNARF™-4F (Thermo Fisher Scientific) at a concentration of 100 µM was used as the inner core solution. PoSo-SNARF were made for optical properties studies with pH_in_ = pH_out_.

### PoSo purification

PoSo purification consisted of removal of the non-encapsulated dye and buffer exchange of the outer medium. The purification was performed by group separation in PD-10 desalting columns (Cytiva, UK), adapting the manufacturer’s gravity protocol as detailed in the Supplementary Methods.

### Polymer quantification in PoSo suspensions

The quantification of the polymer in PoSo samples relied on the absorbance of polystyrene at 270 nm as detailed in the Supplementary Methods.

### Dye quantification in PoSo suspensions

The dye quantification in PoSo samples was based on fluorescence measurements of appropriate calibration standards as detailed in the Supplementary Methods.

### PoSo size measurements

The PoSo size was measured by laser diffraction (Mastersizer 2000, Malvern Instruments, UK) in ultrapure water with the following settings: refractive indices for sample and water: 1.55 and 1.33, respectively; background and sample measurement time: 30 s each; number of measurements: 3; delay between measurements: 2 s; stirrer speed: 3500 rpm. The sample volume was adjusted so that an obscuration > 0.5% was achieved. PS microparticles of 7 μm average diameter (Sigma 78462, Merck) were used as a reference sample.

### Fluorescence of free HPTS vs. PoSo-HPTS

HPTS fluorescence excitation spectra were measured in a 96-well black microplate (polypropylene, f-bottom, Greiner Bio-One) using the Infinite^®^ M200 Pro plate reader (Tecan). Wells were loaded with 250 µL, [HPTS]_well_ = 2 µM for the dye in solution and with 200 µL, [HPTS]_well_ ≈ 5 µM for the PoSo-HPTS.

### PoSo-based ammonia assay

Calibration standards and quality control (QC) solutions of ammonia were prepared by dilution in phosphate buffered saline (PBS, Invitrogen, Thermo Fisher Scientific, MA, USA) of a 0.1 M NH_4_Cl_(aq)_ analytical standard solution for ion-selective electrodes (Sigma 09683, Merck). Calibrants and QCs were prepared independently: two separate stock solutions of 1000 μM NH_4_^+^_(aq)_ in PBS were prepared and further diluted, one of them used for the calibrants and the other one for QCs. For each calibrant level, one solution was prepared but measured in replicate wells. Pure PBS served as blank. For each QC level, replicate solutions were independently prepared.

For the assay, an aliquot of *z* μL of calibrant, QC, blank, or human plasma sample was mixed in a 96-well black microplate (PS, f-bottom, Greiner Bio-One) with 100-*z* μL (for PoSo-HPTS) or 200-*z* μL (for PoSo-hCy) of a PoSo master mix using an electronic multichannel pipette (VOYAGER, Integra Biosciences, UK). For a sample volume fraction φ = 40 vol%, 40 μL of sample and 60 μL of PoSo-HPTS master mix were added per well. Once plate loading was completed, incubation took place in the dark at 20–25 °C for 10 min and plate covered with its lid (for PoSo-HPTS) or for 5 min and plate not covered (for PoSo-hCy), unless stated otherwise. These incubation times were selected based on a previous pilot experiment with a similar PoSo-HPTS system, where a low time dependence of the fluorescence intensity ratio was found for incubation times between 5 and 30 min, hence indicating that equilibrium was practically reached within a time period shorter than 5 min. Well-wise fluorescence measurements were then performed with the Infinite^®^ M200 Pro plate reader at λ_exc_ = 416 nm and 455 nm, λ_em_ = 515 nm for PoSo-HPTS or at λ_exc_ = 630 nm, λ_em_ = 682 nm and 712 nm for PoSo-hCy. The master mix was prepared by diluting the PoSo product batch with the outer medium buffer so that the desired final dye or polymer concentration in the wells could be reached ([HPTS]_assay_ = 0.4 or 2.5 μM for PoSo-HPTS, and [Polymer]_assay_ = 1.3 mg/mL for PoSo-hCy).

### Human plasma

Human plasma was purchased from BioIVT (UK) with the following characteristics: healthy donors, pooled gender, tested negative against several pathogens including hepatitis virus, K_2_EDTA anticoagulant collection, aliquoted after collection, frozen at − 80 °C, shipped on dry ice, and stored immediately at − 80 °C once received to prevent any freeze–thaw cycles. On the day of the experiment, an aliquot was thawed on ice and experiments were performed within 2.5 h after thawing. Throughout this time, the sample was kept on ice as recommended for ammonia quantification in plasma. The total bilirubin content of the plasma samples was measured with a commercial kit (bilirubin assay kit, LOT# 3305897, Sigma-Aldrich, Merck).

### PocketChem™ blood ammonia analyzer

The PocketChem™ Blood Ammonia Analyzer (Arkray, Japan) was used following the manufacturer’s recommendation in the package insert and Q&A document. Namely, the F-6 mode was used for measurements in plasma instead of the F-mode written on the strips, which is recommended for measurements in blood. In brief, 20 μL of sample were added onto the strip using a micropipette and after 180 s the filter of the strip was removed swiftly. For the measurement to be performed, the strip was inserted into the device and the device lid was closed.

The unspiked plasma aliquot was measured once right after the plasma was thawed and twice at the end of the PoSo ammonia assay experiments (i.e. ~ 1–2 h after thawing) to ensure ammonia levels had not risen within the course of the experiment. Ammonia-spiked plasma samples were measured in duplicate.

### Interference testing

The amino acids selected to be tested as potential interferents for the PoSo-HPTS ammonia assay were: l-lysine monohydrochloride (Lys; 99.5%), l-methionine (Met; 99.5%) and l-phenylalanine (Phe; 99.0%), all obtained from Merck. Sodium pyruvate (Pyr; for analysis, AppliChem, Germany) and bilirubin conjugate, ditaurate, disodium salt (Bil; Calbiochem, Merck) were also tested.

In the initial interference evaluation, [HPTS]_in_ = 1 mM, [HPTS]_well_ = 0.4 µM, pH_in_ 6.0, pH_out_ 8.2, and φ = 40 vol% were used. In a typical experiment, a stock solution of the interferent was prepared at 10 mM in PBS. Two NH_4_Cl_(aq)_ stock solutions at 1000 and 2000 µM were also prepared in PBS. An appropriate amount of interferent stock and NH_4_^+^_(aq)_ stock were mixed to provide the desired interferent and ammonia concentrations to be tested. Amino acids and Pyr concentrations of 100, 500, 1000, and 5000 µM were tested in combination with an ammonia concentration ([NH_4_^+^_(aq)_]) of 30, 40, 150, 400, and 750 µM. The remaining volume was adjusted with PBS. Interferent-free ammonia QC solutions were also added to the same microplate. The synthetic analogue of bilirubin (i.e. bilirubin ditaurate), that was used here to challenge the assay, is a mimic of bilirubin glucoronides as it shares their optical properties and water solubility^34^. Bil concentrations of 10, 20, 50, and 100 µM were used for interference challenge.

In the second interference assessment for Bil, PoSo-HPTS were used in optimized conditions to reduce Bil interference: pH_in_ 6.0, pH_out_ 8.2, φ = 40 vol%, [HPTS]_in_ = 2.5 mM, [HPTS]_assay_ = 2.5 µM, and Bil concentrations of 20, 100, 200 and 500 µM were used.

Finally, using PoSo-hCy, a Bil concentration of 500 µM was tested against a low NH_4_^+^(aq) QC of 40 µM using the following setup: pH_in_ 6.5, pH_out_ 9.5, φ = 40 vol%, [hCy]_in_ = 250 μM, [Polymer]_assay_ = 1.3 mg/mL, 200 μL/well.

### Data analysis

Linear or sigmoidal analysis were performed with OriginPro^®^ 2019 (OriginLab, MA, USA). The sigmoidal regression function was a four-parameter logistic function (“logistic” function in OriginPro^®^ 2019) described by the following equation (Eq. 1):

1 $$y={A}_{2}+\frac{{A}_{1}-{A}_{2}}{1+{\left(x/{x}_{0}\right)}^{p}},$$

where A_1_ and A_2_ set the upper and lower limit of the function, respectively, x_0_ is the center, and p is the power. The coefficient of determination was represented by the adjusted R^2^ (Adj. R^2^) calculated with OriginPro^®^ 2019. Back-calculated concentrations of calibrants or QCs were determined directly using the “Find X from Y” feature in OriginPro^®^ 2019 (OriginLab).

In interference analysis experiments, Brown-Forsythe and Welch ANOVA (parametric; unmatched) statistical analysis was performed with Holm-Sidak’s multiple comparison tests using GraphPad Prism 8.2 (GraphPad Software, CA, USA).

The number of independent experiments is denoted as N and the number of technical replicates is denoted as n. The latter is also used for the number of ammonia measurements of independently prepared replicate QC solutions within one experimental run.

## Results and discussion

### PoSo characterization

PoSo of PS block 3500–4500 kDa were used in this study since they were already found well-suited in previous experiments^14,35^. The PoSo diameter distributions by laser diffraction were primarily unimodal with a main mode diameter of 3.5 µm (Supplementary Fig. S1).

### Optical properties of PoSo-HPTS

The propensity of HPTS to self-quench was assessed (Supplementary Fig. S2) and an [HPTS]_in_ of 1 mM was initially selected for the produced PoSo-HPTS. The optical properties of HPTS upon encapsulation into PoSo were then investigated by preparing a series of PoSo-HPTS suspensions with pH_in_ = pH_out_ (Fig. 1b–d and Supplementary Fig. S3). The excitation spectra of free HPTS in buffer solutions with pH between 4.0 and 8.1 are shown in Fig. 1b. As previously reported^36^, the HPTS fluorescence depends highly on pH at λ_exc_ of 455 nm and pH above 5.7 but is pH-independent at λ_exc_ of 416 nm (isosbestic point) (Fig. 1b). The corresponding spectra for PoSo-HPTS with pH_in_ = pH_out_ as well as one case of pH_in_ ≠ pH_out_ (for comparison) are shown in Fig. 1c. The isosbestic point and the λ_exc_ of maximum pH-dependency of HPTS remained almost unaffected by the encapsulation into PoSo (Fig. 1c). Only a slight increase in the onset of the pH-dependent behavior of HPTS (*ca.* 0.5 pH units) was found for PoSo-HPTS in comparison to free HPTS, which is seen when plotting the 515 nm emission intensity ratios I_455_/I_416_ (where I_455_ is the dye fluorescence intensity with λ_exc_ 455 nm, λ_em_ 515 nm and I_416_ the dye fluorescence intensity with λ_exc_ 416 nm, λ_em_ 515 nm) against pH (free HPTS) or pH_in_ (PoSo-HPTS) (Fig. 1d).

A sigmoidal dependence of the I_455_/I_416_ ratio as a function of pH was observed for both the free and PoSo-encapsulated HPTS (Fig. 1d). This is in line with the literature for HPTS^36,37^ and other pH-sensitive fluorescent dyes^38–40^, and originates from the acid/base equilibrium of such dyes that is described by the Henderson–Hasselbalch equation^41^. This dependence was well described by an empirical four-parameter logistic model, as shown in Fig. 1d. Importantly, establishing a pH gradient across the vesicle membrane did not affect the characteristic ratio I_455_/I_416_ used for pH sensing (Fig. 1d, filled triangle *vs.* circle for pH_in_ 5.7).

### Accuracy and precision of PoSo-HPTS ammonia assay

For ammonia sensing, a higher pH_out_ than pH_in_ is necessary to achieve and sustain a concentration gradient of uncharged ammonia molecules across the vesicle membrane^14^. The uncharged ammonia molecules can diffuse from the outer to the inner medium, where they become protonated to ammonium and stably trapped, leading to a pH increase in the PoSo core until equilibrium is established^14^ (Fig. 1a). The initial pH_in_ of the PoSo-HPTS should thus be close to the onset of the pH-dependency of the sensing fluorescence ratio I_455_/I_416_, i.e. in the pH range 5.7–6.2 (Fig. 1d), so that the dynamic increase of pH_in_ upon PoSo-HPTS incubation with an ammonia-containing sample can be directly translated into an increase of the assay signal I_455_/I_416_.

In a previous pilot experiment with a similar PoSo-HPTS system, a pH_in_ of 5.7 and a pH_out_ of 7.4 were used, while linear regression was applied to model the ammonia sensing calibration curve^14^. These conditions were also employed here for comparison (with a sample volume fraction (φ) of 25 vol% in the assay), and a calibration curve of excellent linearity was obtained (Supplementary Fig. S4), as indicated by the high coefficient of determination (Adj. R^2^ of 0.996). PBS was used as a surrogate matrix for the preparation of the calibrant solutions due to the presence of endogenous ammonia in blood plasma.

**Figure 2:**
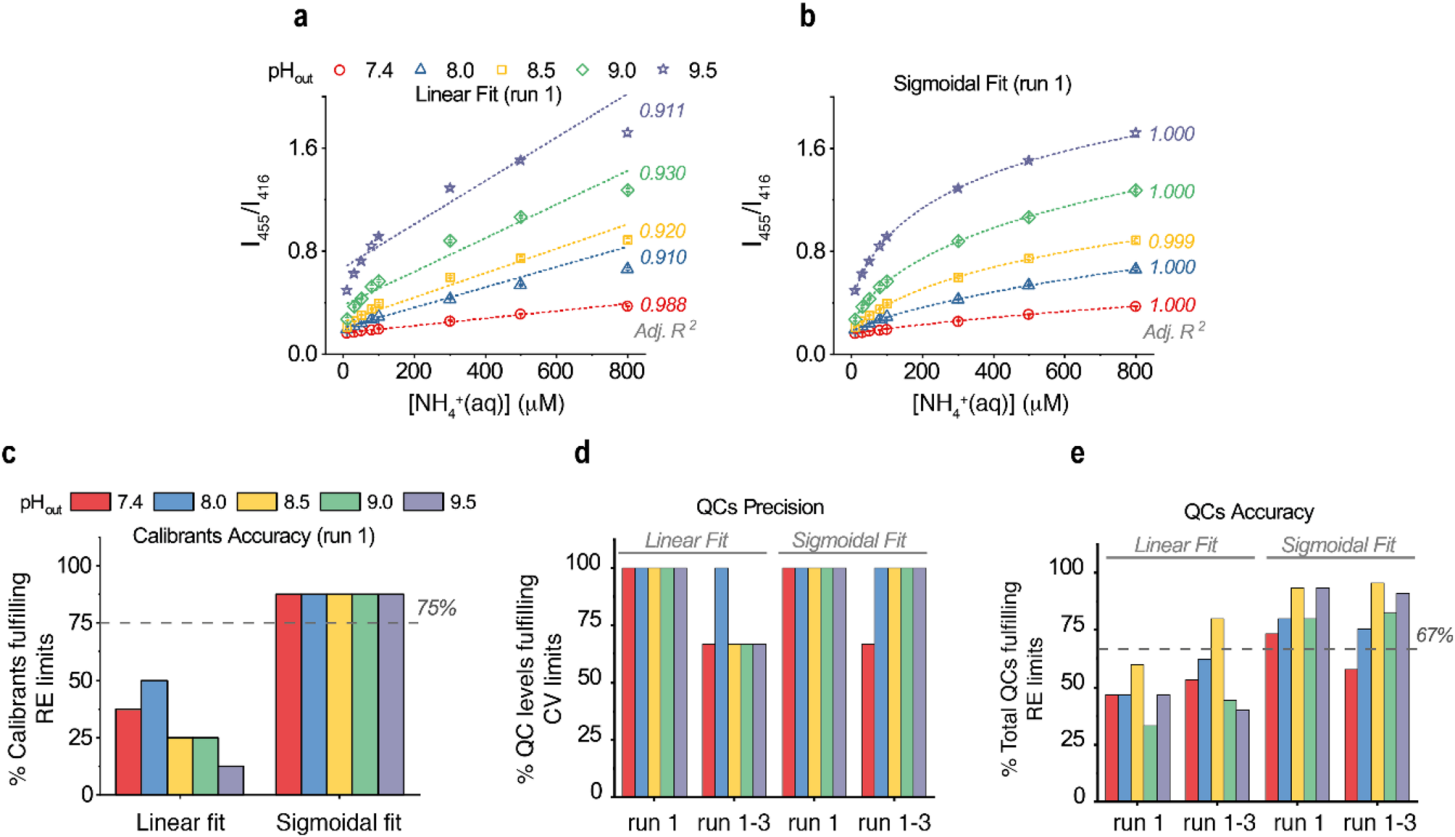
Performance of regression, accuracy, and precision of the PoSo-HPTS ammonia assay using varying pH_out_. (**a–b**) Measured fluorescence intensity ratios I_455_/I_416_ (λ_em_ = 515 nm) (symbols) as a function of [NH_4_^+^_(aq)_] in calibrant solutions (10–800 μΜ in PBS) using PoSo-HPTS with pH_in_ 6.2 and various pH_out_. Calibration curves and coefficients of determination (Adj. R^2^) were obtained with either linear (**a**) or sigmoidal (**b**) regression. For each pH_out_, results are presented as mean ± SD (n = 3) of replicate wells from one experimental run (run 1). In total three independent runs were performed (runs 1, 2, and 3). Conditions: [HPTS]_in_ = 1 mM, [HPTS]_assay_ = 0.4 µM, φ = 25 vol%. (**c**) Fraction of calibration levels from (**a**,**b**) with mean back-calculated [NH_4_^+^_(aq)_] having RE within ± 15% (or ± 20% for LLOQ). Dotted line indicates the minimum acceptable fraction according to EMA^24^ and FDA^23^. (**d**) Fraction of QC levels (60, 400, and 750 μM NH_4_^+^_(aq)_ in PBS; n = 5 independent replicates per level) with assay-determined [NH_4_^+^_(aq)_] having CV within ± 15%. (**e**) Fraction of total QCs (3 levels, n = 5 independent replicates per level) with assay-determined [NH_4_^+^_(aq)_] having RE within ± 15%. Dotted line in (**e**) indicates the minimum acceptable fraction according to EMA^24^ and FDA^23^.

Next, it was investigated whether we could increase the slope of the calibration curve by increasing the transmembrane pH gradient. Therefore, we tuned the pH_out_ between 7.4 and 9.5 while keeping pH_in_ at 6.2 (Fig. 2) or 5.7 (Supplementary Fig. S4). Higher transmembrane pH gradients led indeed to calibration curves of steeper slopes for both pH_in_ values and hence to a wider quantitative detection range, albeit with a concomitant deterioration of linearity (Fig. 2a and Supplementary Fig. S4). The observed non-linear relationship between the I_455_/I_416_ and the [NH_4_^+^]_(aq)_ for pH_out_ above 7.4 (Fig. 2a) is more complex than the relationship between the I_455_/I_416_ and pH_in_ (Fig. 1d) and cannot be merely attributed to the acid/base equilibrium of HPTS. An empirical sigmoidal, four-parameter logistic fit was thus applied and proven as an excellent regression model for the ammonia sensing calibration curves at all pH_out_ values tested (including 7.4), as shown in Fig. 2b and Supplementary Fig. S4.

The linear model could adequately describe the calibration curve of the assay only at the lowest pH_out_ of 7.4 (Fig. 2a). In this case, due to the relatively low fraction of uncharged NH_3_ molecules in the outer medium^42^, the influx of NH_3_ into the PoSo and the associated increase of pH_in_ are relatively small. This likely corresponds to an accordingly small and relatively flat section of the sigmoidal pH-dependency curve of the dye response (Fig. 1d), resulting in a seemingly linear relationship between I_455_/I_416_ and [NH_4_^+^]_(aq)_ in Fig. 2a.

The impact of choosing a sigmoidal instead of a linear fit on the assay performance was further investigated by means of accuracy and precision. The relative error (RE) of the assay-determined *vs.* nominal [NH_4_^+^_(aq)_] served as a measure of accuracy, while the coefficient of variation (CV) reflected the precision (low RE and CV represent high accuracy and precision, respectively). Regarding the calibration curve itself, the guidelines on bioanalytical method validation by EMA^24^ and FDA^23^ suggest that the back-calculated calibrant concentrations should have RE within ± 15%, except for the lower limit of quantification (LLOQ) for which RE up to ± 20% is acceptable, and this should hold true for at least 75% of the calibration levels. It is noted that for ligand-binding assays, which often have sigmoidal calibration curves, the corresponding RE limits by EMA are ± 20% or ± 25%, while two calibration levels can be added [one below the LLOQ and one above the upper limit of quantification (ULOQ)] to serve as “anchor points” that do not require acceptance criteria. However, in Fig. 2, all 8 calibration levels from 10 to 800 µM NH_4_^+^_(aq)_ in PBS were considered as calibrants (no anchor point) and the more stringent RE limits (± 15 or ± 20%) were applied for both fits.

Figure 2c shows the fraction of the calibration levels from Fig. 2a,b whose mean (n = 3 wells per level) back-calculated [NH_4_^+^_(aq)_] complied with the aforementioned EMA criteria. When linear fit was applied, no pH_out_ led to more than 75% of calibration levels with acceptable accuracy (Fig. 2c). On the contrary, sigmoidal fit resulted in calibration curves with ≥ 75% calibrants with acceptable accuracy for all pH_out_ values (Fig. 2c). For the sake of clarity, Fig. 2a–c shows the results for one experiment (N = 1), though two more independent experiments (or “runs”) were performed on consecutive days for all conditions. In the two new runs, acceptable accuracy was again only observed for < 75% of calibrants when using linear fit. However, using a sigmoidal fit, > 75% of calibrants showed acceptable accuracy in these new runs for all pH_out_ except pH_out_ 7.4 (here only 63% of calibrants had acceptable accuracy).

In every independent run, QC solutions in PBS were assessed at three [NH_4_^+^_(aq)_] levels (60, 400, and 750 μM), with five independently prepared replicate solutions (n = 5) per level. The precision and accuracy of the assay-determined [NH_4_^+^_(aq)_] were compared between linear and sigmoidal fit using data from each run (N = 1) or from all three runs (N = 3) to express the within- or between-run precision and accuracy. Excellent within-run precision was obtained for both regression models in run 1 (Supplementary Table S1), and this was generally also the case for runs 2 and 3 (except for two cases with the linear fit). In terms of between-run precision (Supplementary Table S1), however, the linear fit resulted in CV ≤ 15% only for the QCs of 400 and 750 μM, while for the lowest QC of 60 μM a CV > 15% was observed for almost all pH_out_ (except pH_out_ 8.0). The sigmoidal fit, on the other hand, led to excellent between-run precision in all cases, except for the isolated case of 60 μM QC at pH_out_ 7.4 where a CV of 17% was observed (Supplementary Table S1). The superior between-run precision with sigmoidal fitting is also illustrated in Fig. 2d, where the fraction of QC levels with CV ≤ 15% is shown for run 1 (within-run precision) and runs 1–3 (between-run precision).

In terms of accuracy, the mean RE was often > 15% for the linear fit both within-run and between-run, in contrast to the sigmoidal fit for which RE ≤ 15% was observed in the majority of cases (Supplementary Table S1). For a better overview of the assay accuracy, Fig. 2e shows the fraction of total QCs (3 levels, 5 independent replicates per level) with acceptable accuracy in run 1 (N = 1) or all three runs (N = 3) for all pH_out_ values and both types of fit. These fractions are compared with a limit of 67% (dotted line in Fig. 2e), in accordance with the EMA^24^ and FDA^23^ guidelines. In the case of linear fit, no tested pH_out_ value led to ≥ 67% of total QCs with acceptable accuracy in run 1 while only for pH_out_ 8.5 this limit was surpassed in the between-run evaluation. On the contrary, all pH_out_ values resulted in ≥ 67% of total QCs with acceptable accuracy for the sigmoidal fit, with the only exception being pH_out_ 7.4 in the between-run evaluation for which a fraction of 58% was observed.

Taken together, the sigmoidal fit outperformed the linear one in respect of coefficient of determination, accuracy, and precision. Therefore, the former was used for further optimization of the ammonia assay. Regarding pH_out_ selection, pH_out_ 7.4 was excluded from further studies as it yielded the lowest accuracy and precision for sigmoidal calibration curves. Furthermore, pH_out_ values > 8.5 were excluded and a refinement in the range 8.0–8.5 was performed in order to maintain a high assay performance while minimizing the amplitude of the transmembrane pH-gradient. Considering our results at pH_in_ 5.7 and 6.2, pH_in_ 6.0 was selected for all further experiments.

**Figure 3:**
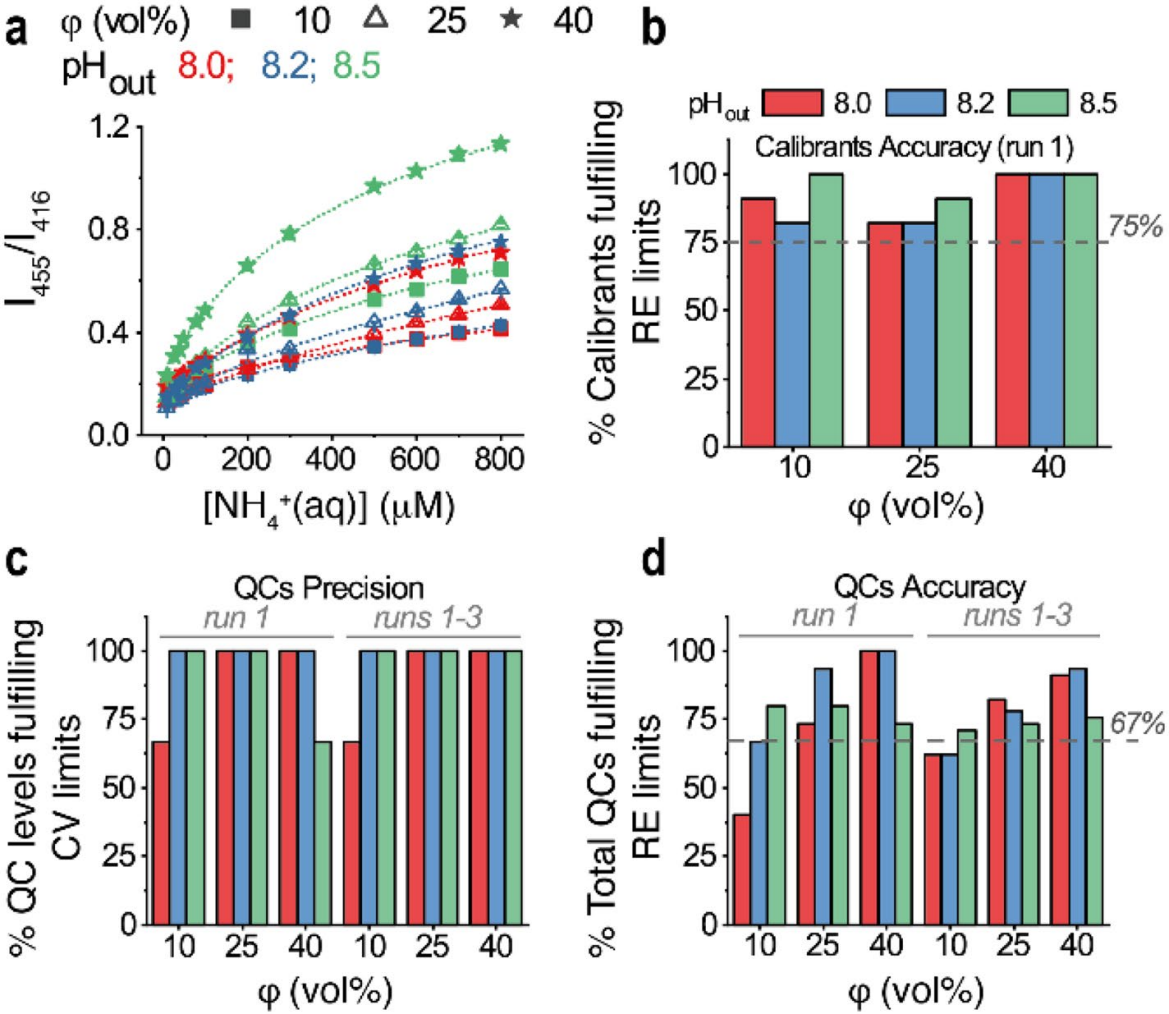
PoSo-HPTS calibration curves and assay performance for varying φ and pH_out_. (**a**) Ammonia sensing calibration curves obtained with sigmoidal regression fit of the measured fluorescence intensity ratio I_455_/I_416_ (λ_em_ = 515 nm) data plotted over [NH_4_^+^_(aq)_] in the calibrant solutions. Assay was performed at various φ and pH_out_ values using PoSo-HPTS. Eleven calibration levels within 30–800 µM NH_4_^+^_(aq)_ in PBS were used, while a 10 μM standard served as anchor point. For each condition, results are presented as mean ± SD (n = 3) of replicate wells from one experimental run (run 1); error bars may be smaller than symbols. In total three independent runs were performed (runs 1, 2, and 3). Conditions: pH_in_ 6.0, [HPTS]_in_ = 1 mM, [HPTS]_assay_ = 0.4 µM. (**b**) Fraction of calibration levels from (**a**) with mean back-calculated [NH_4_^+^_(aq)_] having RE within ± 15% (or ± 20% for LLOQ). Dotted line indicates the minimum acceptable fraction according to EMA^24^ and FDA^23^. (**c**) Fraction of independently prepared QC replicates (n = 5) per level (45, 400, and 750 μM NH_4_^+^_(aq)_ in PBS; corresponding to φ = 10, 25, and 40 vol%, respectively) with assay-determined [NH_4_^+^_(aq)_] having CV within ± 15% (or ± 20% for the lowest QC). (**d**) Fraction of total QCs (3 levels, n = 5 independent replicates per level) with assay-determined [NH_4_^+^_(aq)_] having RE within ± 15% (or ± 20% for the lowest QC). Dotted line in (**d**) indicates the minimum acceptable fraction in accordance with EMA^24^ and FDA^23^.

Furthermore, the effect of the amount of ammonia molecules relative to PoSo on assay performance was investigated by varying the volume fraction (φ) of the ammonia-containing sample in the assay while keeping a constant PoSo-HPTS concentration. Figure 3a shows ammonia assay calibration curves obtained with PoSo-HPTS and performed with φ = 10, 25, or 40 vol% and pH_out_ 8.0, 8.2, or 8.5. Eleven calibration levels in the range 30–800 µM NH_4_^+^_(aq)_ in PBS were used, while a 10 μM standard served as anchor point. For all pH_out_ values, increasing φ resulted in steeper calibration curves (and thus enhanced sensitivity) in all three independently performed experimental runs (Fig. 3a shows results for run 1). This can be attributed to the increasing amount of ammonia relative to PoSo, which leads to a higher influx of uncharged NH_3_ into the vesicle core and thereby a larger increase in pH_in_ for a given [NH_4_^+^_(aq)_] in the calibrant solutions. The Adj. R^2^ was ≥ 0.995 for all calibration curves, further confirming the robustness of the four-parameter logistic model for the assay. All calibration curves of run 1 resulted in ≥ 75% of calibration levels with RE of the mean back-calculated [NH_4_^+^_(aq)_] being within ± 15% (or ± 20% for the LLOQ) (Fig. 3b), similarly to runs 2 and 3.

QCs prepared in PBS were used at three [NH_4_^+^_(aq)_] levels (45, 400, and 750 μM), with five independently prepared replicate solutions (n = 5) per level. Excellent within- and between-run precision was observed for nearly all φ and pH_out_ values tested (Fig. 3c and Supplementary Table S2). In terms of accuracy, a general trend of improved performance with increasing φ from 10 to 40 vol% was noted, especially for pH_out_ 8.0 and 8.2 (Fig. 3d and Supplementary Table S2). The highest within-run (run 1) and between-run (runs 1–3) accuracy was obtained for the combination φ = 40 vol% and pH_out_ 8.2, which was hence selected for subsequent experiments. Overall, pH_out_ and ϕ were the most impactful parameters to increase the assay response span and thus in turn, its sensitivity, accuracy, precision and ammonia quantification range.

To further improve assay performance, the calibration curve was extended to include an additional anchor point at 1000 μM NH_4_^+^_(aq)_, besides the already applied low anchor at 10 μM. This modification yielded a sensing range between 30 and 800 μM NH_4_^+^_(aq)_, which is adequate for ammonia sensing in plasma, as it covers patients with normal and elevated ammonia levels^5,7^. To validate the assay, five (N = 5) independent runs were performed over five consecutive days. The obtained calibration curves are shown in Supplementary Fig. S5a, indicating an excellent between-run reproducibility. In all runs, the Adj. R^2^ was ≥ 0.999 and 100% of the calibration levels passed the RE limits (Supplementary Fig. S5), highlighting the benefit of using two anchor points. Upon QCs preparation in PBS, the EMA^24^ and FDA^23^ guidelines were followed for the selection of the [NH_4_^+^_(aq)_] levels, namely we included a QC at the LLOQ, which should be identical to the lowest calibrant (i.e. 30 μM), one within three times the LLOQ (low QC, which was set to 40 μM), one at around 50% of the calibration curve range (medium QC, set to 400 μM), and one at ≥ 75% of the upper calibration curve range (high QC, set to 750 μM). Additionally, a QC at 150 μM was used. In each level, five replicate solutions (n = 5) were prepared independently. As shown in Supplementary Fig. S5b,c, both the within-run (run 1) and between-run (runs 1–5) accuracy and precision requirements were surpassed, as 100% of the QC replicates per level and hence 100% of the total QCs had RE and CV within ± 15% (or ± 20% for the LLOQ). As a further indication of robustness of the assay on achieving an LLOQ of 30 μM, the assay-determined values for all replicates of the 30 μM QC are shown in Supplementary Fig. S5d for run 1 and all five runs. When all runs are taken into account, the values are randomly scattered around a mean value of 31 μM within the limiting RE of ± 20%.

Overall, these data confirmed that the optimized PoSo-HPTS ammonia assay performed at a regulatory acceptable standard for ammonia quantification within 30–800 µM, when assessed in PBS. The optimized PoSo-HPTS properties and assay conditions were defined as: 5.5 mM citric acid as inner buffer at pH 6.0 and 300 mOsm/kg, 50 mM borate as outer buffer at pH 8.2 and 300 mOsm/kg, [HPTS]_in_ = 1 mM, [HPTS]_assay_ = 0.4 µM, φ = 40 vol% with a total volume of 100 μL/well in the assay plate, and four-parameter logistic fit for calibration.

### Interference testing

Operating the PoSo-HPTS assay in the aforementioned optimized conditions, we then sought to investigate its performance in the presence of potential endogenous interferents. Each interferent was added at various concentrations to NH_4_^+^_(aq)_ control solutions in PBS and the assay-determined mean ammonia concentrations in the presence and absence of interferent were compared.

**Figure 4:**
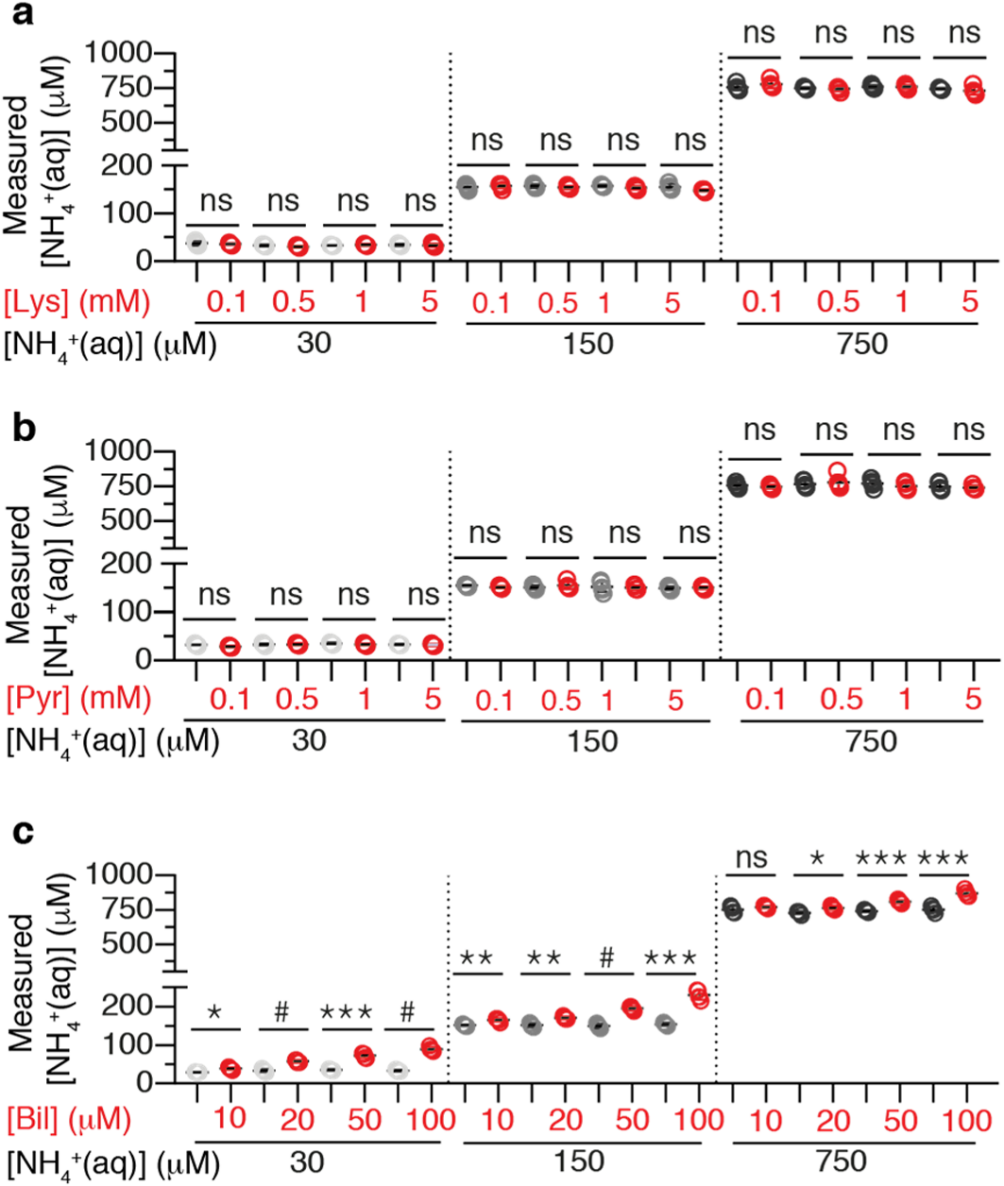
Analytical selectivity screening. Measured [NH_4_^+^_(aq)_] in the absence (grey scale symbols) and presence (red symbols) of Lys (**a**), Pyr (**b**), and Bil (**c**) at various concentrations in solutions containing 30, 150, or 750 µM of NH_4_^+^_(aq)_ in PBS. Results are shown as exact measured values and mean ± SD (n = 5 independently prepared QC replicates with or without interferent). Conditions: pH_in_ 6.0, pH_out_ 8.2, φ = 40 vol%, [HPTS]_in_ = 1 mM, [HPTS]_assay_ = 0.4 µM. For each interferent, Brown-Forsythe and Welch ANOVA (parametric; unmatched) statistical analysis was performed with Holm-Sidak’s multiple comparison tests, where ns, *, **, *** and # correspond to p > 0.05, p ≤ 0.05, p ≤ 0.01, p ≤ 0.001 and and p ≤ 0.0001, respectively.

Amino acids were selected among other endogenous compounds in plasma to assess the selectivity of PoSo towards ammonia in the presence of amine-containing molecules. Each of the three selected amino acids had very different structures, thereby representing a different possible interaction with the PoSo surface. These included the positively charged (basic) Lys, the neutral and nonpolar Met, and the aromatic Phe. These amino acids were tested in a broad concentration range that spanned below and above their physiological plasmatic levels^26^. Statistically non-significant (ns) differences (p > 0.05) between samples with and without amino acids were obtained, indicating that all three amino acids do not interfere with the assay (Fig. 4a and Supplementary Fig. S6). This is consistent with a previous work which used a similar PoSo-HPTS assay that also showed to be selective towards ammonia in the presence of a variety of amino acids and other amine-containing molecules^14^.

Pyr was included in the interference study as it is a common interferent for enzymatic ammonia assays^27^. The employed concentration range in this case embraced the threshold value of 750 μM, which is reported by several manufacturers of enzymatic, in vitro diagnostic tests for ammonia [e.g. Ammonia L3K^®^ Assay (Sekisui Diagnostics), Infinity™ Ammonia Reagent (Fisher Diagnostics/Beckman Coulter Inc.), or Ammonia Ultra (SENTINEL CH. SpA/Abbott Laboratories Inc.)] as the maximum plasma Pyr concentration for no interference. As expected, Pyr did not interfere with the assay even at concentrations as high as 5000 μM (Fig. 4b and Supplementary Fig. S6).

**Figure 5:**
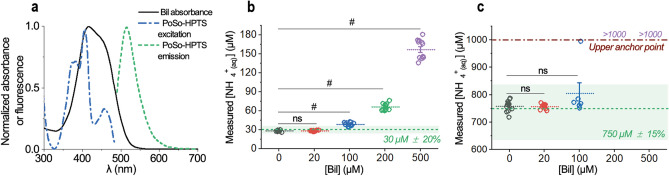
Reducing Bil interference in the PoSo-HPTS ammonia assay. (**a**) Normalized spectra of Bil absorbance and PoSo-HPTS fluorescence excitation (λ_em_ = 515 nm) and emission (λ_exc_ = 455 nm) spectra. (**b–c**) Measured [NH_4_^+^_(aq)_] in the absence and presence of various Bil concentrations in solutions containing 30 μM (**b**) or 750 μM (**c**) NH_4_^+^_(aq)_ in PBS using PoSo-HPTS with adjusted conditions to reduce Bil interference (i.e. pH_in_ 6.0, pH_out_ 8.2, φ = 40 vol%, [HPTS]_in_ = 2.5 mM, [HPTS]_assay_ = 2.5 µM). Results are shown as exact measured values and mean ± SD. The nominal [NH_4_^+^_(aq)_] with ± 20% (**b**) or ± 15% (**c**) RE is depicted as green dashed line and shaded area. Assay-determined [NH_4_^+^_(aq)_] beyond the upper anchor point are indicated as “> 1000”. Brown-Forsythe and Welch ANOVA (parametric; unmatched) statistical analysis was performed with Holm-Sidak’s multiple comparison tests, where ns and # correspond to p > 0.05 and p ≤ 0.0001, respectively.

Bil was also tested since bioanalytical assays developed for blood or plasma are desired to cope with values of bilirubin at least up to 500 µM^28^, which accounts for the potentially elevated bilirubin levels in the plasma of patients with liver diseases^43^. However, Bil interfered with the ammonia measurements at all challenge concentrations (Fig. 4c and Supplementary Fig. S6). The interference of Bil is optical and can be attributed to the overlap between the Bil absorbance spectrum and the PoSo-HPTS fluorescence spectra (Fig. 5a). This means that when fluorescence measurements for the PoSo-HPTS assay are performed at λ_exc_ = 416 nm and 455 nm with λ_em_ = 515 nm, part of the incident and emitted photons are absorbed by bilirubin, hence altering the PoSo-HPTS fluorescence signal compared to the signal that would be obtained for the same ammonia concentration in the absence of bilirubin (calibration is performed in bilirubin-free solutions).

We therefore tried to reduce the optical interference of Bil in the PoSo-HPTS assay by increasing the [HPTS]_assay_. An increased total HPTS concentration in the produced batches was thus required, and to achieve this, various process parameters including the [HPTS]_in_ as well as the injected polymer solution volume during PoSo preparation were tuned. It was found that the most effective reduction of Bil interference could be achieved using a PoSo-HPTS batch with a [HPTS]_in_ = 2.5 mM (instead of 1 mM) and tested with a [HPTS]_assay_ = 2.5 μM (instead of 0.4 μΜ), keeping all other conditions unchanged (i.e. pH_in_ 6.0, pH_out_ 8.2, φ = 40 vol%). It is noted that also with these modified PoSo-HPTS, 100% of calibrants and QCs fulfilled the required accuracy and precision criteria for ammonia quantification in aqueous buffers (no interferent present) (Supplementary Fig. S7).

The [NH_4_^+^_(aq)_] measured with the modified PoSo-HPTS assay in the presence of various Bil concentrations is shown in Fig. 5b,c. The higher [HPTS]_assay_ and thus HPTS signal in the new setup alleviated the interference of bilirubin, and the assay could retrieve the nominal [NH_4_^+^_(aq)_] accurately for [Bil] up to at least 20 μM. At [Bil] of 100 μM or higher, however, Bil started interfering again with the assay. With this level of tolerance for Bil, our assay using PoSo-HPTS is in principle appropriate for NH_4_^+^_(aq)_ measurements in healthy human plasma (physiological bilirubin concentrations up to ~ 20 μM^44^) and in plasma samples of individuals with slightly elevated bilirubin levels.

### PoSo-HPTS assay for ammonia measurements in human plasma

**Figure 6:**
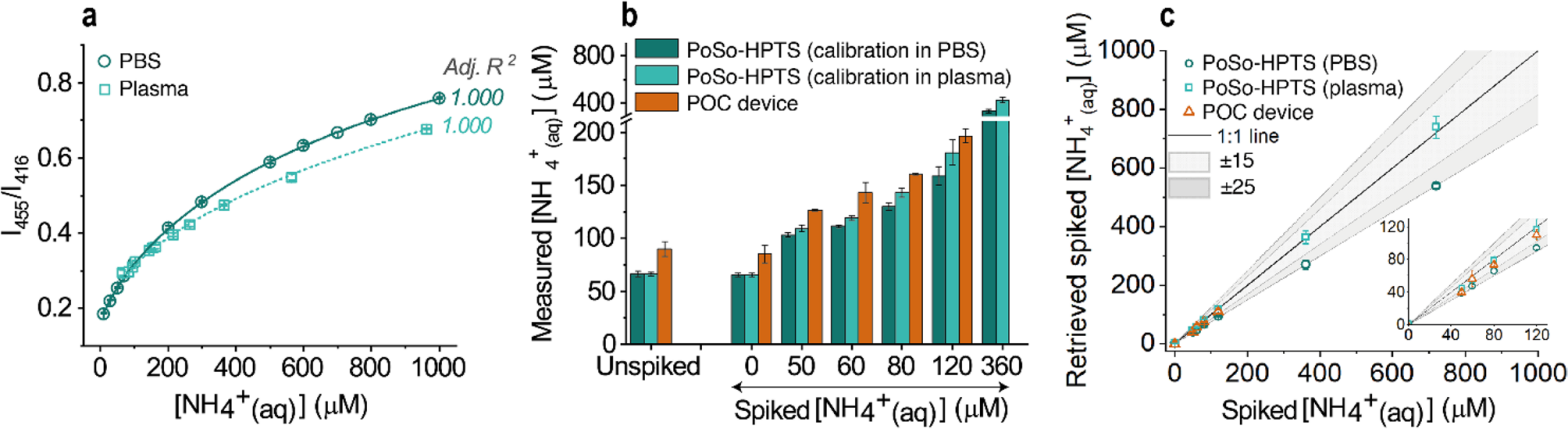
Ammonia measurements in plasma with PoSo-HPTS and a POC device. (**a**) Ammonia sensing calibration curves obtained with sigmoidal regression fit of the measured fluorescence intensity ratio I_455_/I_416_ (λ_em_ = 515 nm) data plotted over [NH_4_^+^_(aq)_] in the calibrant solutions which were prepared either in PBS or in healthy human plasma. Results correspond to mean ± SD (n = 3) of replicate wells. Conditions: pH_in_ 6.0, pH_out_ 8.2, φ = 40 vol%, [HPTS]_in_ = 2.5 mM, [HPTS]_assay_ = 2.5 µM. (**b**) [NH_4_^+^_(aq)_] in unspiked and ammonia-spiked healthy human plasma samples as measured by the PoSo-HPTS assay using the calibration curves from (**a**) and by the POC device. Results correspond to mean ± SD [n = 4 independent replicate solutions (PoSo-HPTS); n = 2 independent replicate solutions (POC device)]. (**c**) Retrieved spiked [NH_4_^+^_(aq)_] from (**b**) as a function of the nominal spiked [NH_4_^+^_(aq)_]. The “1:1” black line corresponds to the ideal case where the retrieved spiked [NH_4_^+^_(aq)_] equals the nominal one and grey areas indicate the corresponding ± 15% and ± 25% RE. Inset shows the same data but only up to 120 μM NH_4_^+^_(aq)_. Mean ± SD [n = 4 (PoSo-hCy); n = 2 (POC)].

The PoSo-HPTS assay using the improved conditions from Fig. 5 was applied for ammonia quantification in human plasma and its performance was compared to the PocketChem Blood Ammonia Analyzer, an in vitro diagnostics POC device that uses colorimetric detection of alkalized ammonia via microdiffusion on a strip^45^. Figure 6a shows the ammonia sensing calibration curves of the PoSo-HPTS assay obtained with calibrant solutions prepared either in PBS or in (ammonia-spiked) healthy human plasma. Both calibration curves had an excellent coefficient of determination and were used to determine the total [NH_4_^+^_(aq)_] in a healthy human plasma sample before and after spiking it with various concentrations of NH_4_^+^_(aq)_ (Fig. 6b). For spiked [NH_4_^+^_(aq)_] up to 120 μM, the measured ammonia concentrations from the PoSo-HPTS assay (using either calibration curve) were comparable to the values received with the POC device (Fig. 6b), with a maximum RE of − 26% for the PoSo-HPTS assay on the unspiked plasma sample and a minimum RE of − 8% for the assay on the 120 μM NH_4_^+^_(aq)_-spiked plasma sample (Supplementary Table S3). It is noted that due to the relatively low ULOQ of the POC device (reportedly 286 μM), this method could not be used on NH_4_^+^_(aq)_-spiked plasma samples examined in this experiment above this range (Fig. 6b).

The PoSo-HPTS assay with calibrants in PBS resulted generally in lower measured [NH_4_^+^_(aq)_] values than that with calibrants in plasma, with the RE increasing for increasing spiked [NH_4_^+^_(aq)_] (Fig. 6b and Supplementary Table S3). This can be explained by the observed concentration-dependent deviation between the two calibration curves (Fig. 6a), which suggests the presence of some matrix effects when PBS is used as calibration medium^46^. Nevertheless, the RE on the [NH_4_^+^_(aq)_] determined by the calibration curve in PBS vs. the one in plasma was lower than 25% (Fig. 6a), and hence PBS could be a reasonable surrogate matrix for plasma. This is also confirmed when considering the retrieved spiked [NH_4_^+^_(aq)_] (Fig. 6c), for which a high accuracy (RE ≤ 25%) was obtained for the PoSo-HPTS assay using either calibration curve (i.e. whether calibrants were prepared in plasma or PBS). The retrieved spiked [NH_4_^+^_(aq)_] values from the PoSo-HPTS assay compared well with the data obtained with the POC device (Fig. 6c).

### Alternative fluorescent dye to overcome bilirubin interference

To overcome the interference from bilirubin, we sought a water-soluble, pH-sensitive, ratiometric fluorophore that absorbs and emits above 550 nm. In terms of optical properties, a molecular dye with excitation and emission in the second near-infrared window (NIR-II) (1000–1700 nm)^47^ would be ideal for increasing the signal to noise ratio in plasma and even working with blood samples. NIR-II fluorophores, however, show an important drop of quantum yield in the presence of water, as they are generally very hydrophobic and have not been made pH-sensitive to our knowledge^47^.

Among the commercially available, ratiometric, pH-sensitive fluorophores with excitation/emission wavelengths longer than those of HPTS, a rhodamine derivative (SNARF™-4F) was the first one to be tested here (Supplementary Fig. S8). Upon excitation at λ_exc_ = 515 nm, SNARF™-4F exhibits an isoemissive point at λ_em_ = 635 nm and a pH-dependent emission peak at λ_em_ = 660 nm (Supplementary Fig. S8). However, the SNARF™-4F fluorescence and pH sensitivity were largely affected by the encapsulation into PoSo (Supplementary Fig. S8). This effect was attributed to a shift of the equilibrium towards the lactone which, in equilibrium with the protonated SNARF™-4F, is devoid of absorbance in the visible region (the cyclization breaks the π network)^48,49^. The lactone is favored by apolar aprotic milieu^48^, hence the altered fluorescence properties upon encapsulation may be an indication of SNARF™-4F partitioning in the PS-*b*-PEG bilayer. Apart from being extensively characterized for fluorophores in the bulk^50^, such alterations of optical properties have been reported upon interaction of (pH-sensitive) fluorophores with other membrane bilayers, such as in liposomes^51,52^.

Wan et al*.*^33^ reported on a pH-sensitive hemicyanine fluorophore (Lyso-pH) that exhibits an isoemissive point at λ_em_ = 670 nm and an emission peak of maximum pH-dependency at λ_em_ = 708 nm (λ_exc_ = 635 nm). This dye was evaluated next and PoSo-Lyso were produced (Supplementary Fig. S9). PoSo-Lyso also exhibited pH-dependency in emission, though in a different pH range (pH 2–6.5) compared to the free Lyso-pH (pH 4–7.5) (Supplementary Fig. S9). Considering the relatively low hydrophilicity of Lyso-pH, this hints to a possible partitioning^51,53^ and/or non-covalent interactions^54,55^ of the dye with the polymeric bilayer, alike SNARF™-4F. Furthermore, for a given pH_in_, the I_670_/I_708_ ratios of PoSo-Lyso made with pH_out_ > pH_in_ were lower than those of PoSo-Lyso made with pH_in_ = pH_out_, which could indicate non-specific binding of the dye to the vesicle membrane and/or dye leakage to the outer PoSo medium. The incorporation of the surfactant SDS in the encapsulated Lyso-pH solution upon PoSo preparation minimized this effect (Supplementary Fig. S9), but the increased complexity of the system led us to set aside this approach.

**Figure 7:**
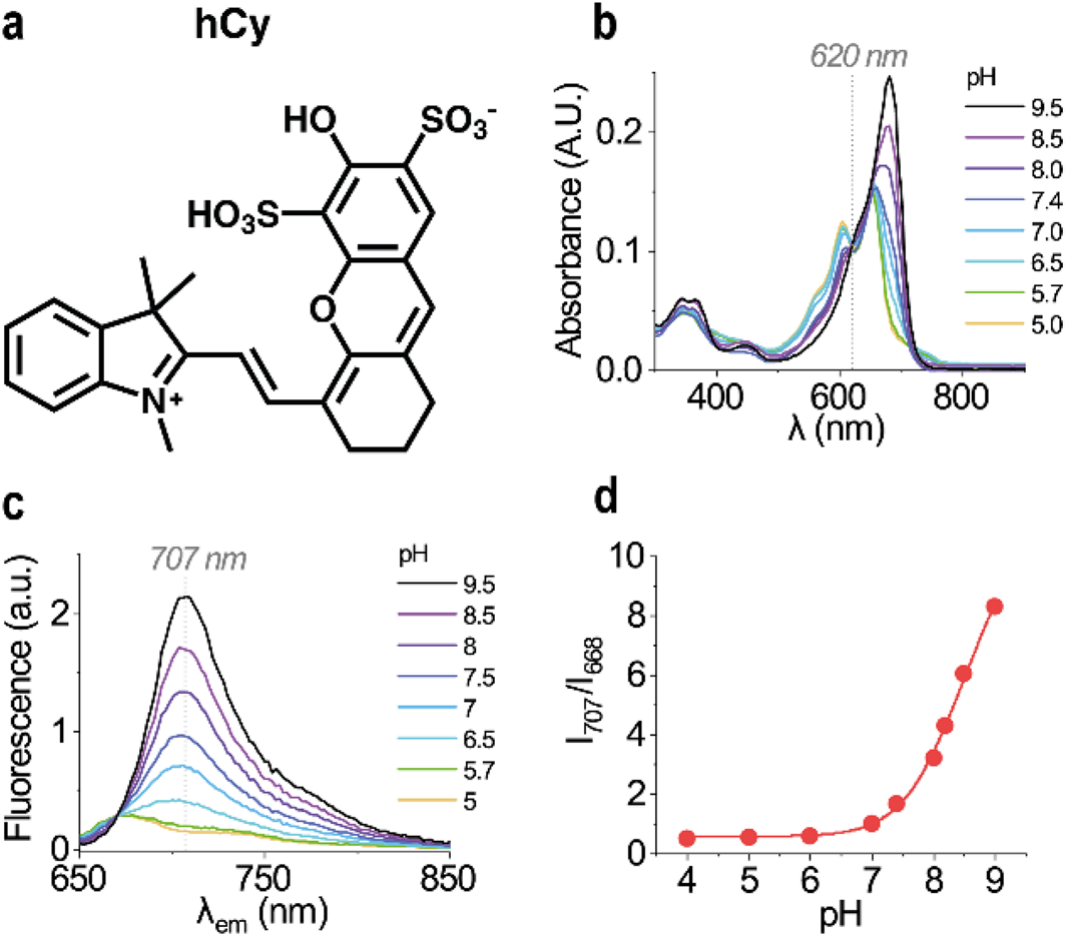
Alternative fluorescent dye to overcome bilirubin interference. (**a**) hCy structure; the product consists of a mixture of disulfonation isomers. (**b**,**c**) Absorbance (**b**) and fluorescence emission (**c**) spectra of free hCy in aqueous solutions of various pH ([hCy] = 10 μM). (**d**) Fluorescence intensity ratio I_707_/I_668_ (λ_exc_ = 630 nm) as a function of pH for free hCy.

Finally, a hemicyanine dye (hCy) that was reported by Yuan et al.^32^ (Fig. 7a) had the desired characteristics, and was selected to produce PoSo-hCy for the ammonia assay. The pH-dependency and optical properties of hCy are illustrated in Fig. 7b,c. In absorbance, it exhibits an isosbestic point at ~ 620 nm (Fig. 7b), while an isoemissive point (at ~ 668 nm) and a wavelength of maximum pH-dependency (at ~ 707 nm) are observed in its fluorescence emission spectra (Fig. 7c). Plotting the fluorescence ratio I_707_/I_668_ as a function of pH (Fig. 7d) revealed a sigmoidal pH-dependency at pH above 6.5.

After an initial optimization process, PoSo-hCy for the ammonia assay were made with a pH_in_ of 6.5 and a pH_out_ of 9.5, while the fluorescence measurements in the assay were performed at λ_em_ = 682 nm and 712 nm for λ_exc_ = 630 nm. The employed wavelengths were slightly modified compared to the aforementioned characteristic wavelengths of free hCy, due to the slight shift of the fluorophore’s optical properties after its encapsulation into the vesicles. Furthermore, PoSo-hCy ammonia assays were performed with an incubation time of 5 min to minimize well-to-well cross-talk that was observed when longer incubation times and/or a pH_out_ of 10 were used (Supplementary Fig. S10). This cross-talk was attributed to an interference from wells containing solutions of high [NH_4_^+^_(aq)_] to neighboring wells of relatively low [NH_4_^+^_(aq)_], due to ammonia evaporation from the former and recondensation in the latter (pK_a_ NH_4_^+^/NH_3_ = 9.2).

**Figure 8:**
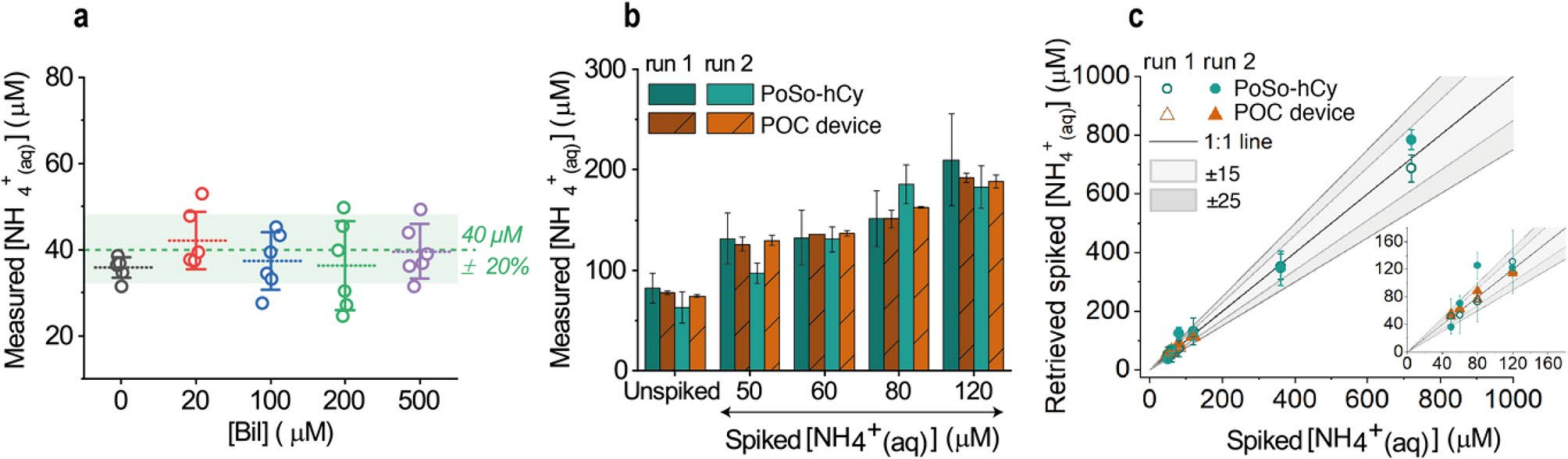
Alleviation of bilirubin interference and ammonia measurements in plasma with PoSo-hCy and a POC device. (**a**) [NH_4_^+^_(aq)_] measured using PoSo-hCy in solutions containing 40 μM NH_4_^+^_(aq)_ and various concentrations of Bil in PBS. Calibration curve from Supplementary Fig S11 was used. Results are shown as exact measured values and mean ± SD (n = 6) of independent replicate solutions. The nominal [NH_4_^+^_(aq)_] with ± 20% RE is depicted as green dashed line and shaded area. Brown-Forsythe and Welch ANOVA (parametric; unmatched) statistical analysis was performed with Holm-Sidak’s multiple comparison tests, indicating non-significant differences compared to the control (p > 0.05) for all [Bil]. (**b**) [NH_4_^+^_(aq)_] in unspiked and ammonia-spiked healthy human plasma samples as measured by the PoSo-hCy assay and the PocketChem device in two independent experiments (runs 1 and 2). For each run, data are presented as mean ± SD [n = 4 independent replicate solutions (PoSo-hCy); n = 2 independent replicate solutions (POC device)]. (**c**) Retrieved spiked [NH_4_^+^_(aq)_] from (**b**) as a function of the nominal spiked [NH_4_^+^_(aq)_]. The “1:1” black line corresponds to the ideal case where the retrieved spiked [NH_4_^+^_(aq)_] equals the nominal one and grey areas indicate the corresponding ± 15% and ± 25% RE. Inset shows the same data but only up to 160 μM NH_4_^+^_(aq)_. Mean ± SD [n = 4 (PoSo-hCy); n = 2 (POC)]. Conditions: pH_in_ 6.5, pH_out_ 9.5, φ = 40 vol%, [hCy]_in_ = 250 μM, [Polymer]_assay_ = 1.3 mg/mL, 200 μL/well.

Performing the PoSo-hCy ammonia assay with calibrant solutions in PBS, a sigmoidal calibration curve with an excellent coefficient of determination was obtained (Supplementary Fig. S11). The [NH_4_^+^_(aq)_] measured for the low QC using the calibration curve from Supplementary Fig. S11 in solutions containing various concentrations of Bil is shown in Fig. 8a, confirming the expected absence of Bil interference when hCy is the assay’s fluorescence reporter. In fact, Bil did not interfere with the assay even with Bil concentrations as high as 500 μM (Fig. 8a), which would in principle allow the quantification of ammonia in plasma samples of patients with liver diseases^5,7^. The accurate ammonia quantification both at lower and higher concentrations of bilirubin with PoSo-hCy is attributed to the absence of an optical interference in this case, as the fluorescence measurements are performed at λ_exc_ = 630 nm with λ_em_ = 682 nm and 712 nm, i.e. at wavelengths with nearly zero optical absorbance for bilirubin (Fig. 5a).

The PoSo-hCy ammonia assay was then applied for ammonia quantification in healthy human plasma samples (unspiked and ammonia-spiked) and was compared to the POC device in two independent experiments (Fig. 8b). The majority of the ammonia concentrations that were measured with the PoSo-hCy assay were comparable with those obtained with the POC device (Supplementary Table S4), though a slightly higher deviation among independent experiments was observed for the former. Furthermore, the retrieved spiked [NH_4_^+^_(aq)_] from the PoSo-hCy assay were highly accurate, with RE ≤ 25% for 83% (or 10 out of 12) data points, comparing well with the POC device (Fig. 8c). Taken together, the use of hCy as an alternative fluorophore for the PoSo-based ammonia assay allowed us to eliminate the interference of Bil while maintaining a high performance in terms of ammonia quantification in a complex biological matrix such as plasma.

## Conclusion

In this work, we developed an analytical assay for the quantification of ammonia in blood plasma. The technology, based on transmembrane pH-gradient PoSo, represents a promising alternative to existing ammonia quantification tests and circumvents part of the limitations commonly encountered in other strategies, such as Pyr interference. Interestingly, the assay presented some of the characteristics of a chromatographic assay and some of a ligand-binding assay, albeit not falling under either category. The use of an empirical four-parameter logistic fitting (similarly to ligand-binding assays) allowed to widen the quantification range to 30–800 µM in surrogate PBS. The assay was further refined to a working range of 40–800 µM in presence of up to 500 µM Bil, with the lower limit of the range being still below the higher end of ammonia levels found in healthy patients. Finally, in human plasma, the assay showed equivalent performance to a gold standard POC device while outperforming the ammonia quantification range of the latter in highly concentrated samples. Throughout process development, the assay was tested and confirmed to comply with regulatory standards to ensure a realistic end product. The PoSo-based ammonia assay herein was shown in a 96-well plate format, suitable for high throughput analysis. Nevertheless, the technology may be converted to provide POC practicality in the future.

## Supplementary Information

Supplementary Information.
