# Supplementary material for: Optimization of an ammonia assay based on transmembrane pH-gradient polymersomes
Source: Sci Rep. 2021 Nov 11;11:22032. doi: 10.1038/s41598-021-01137-1 (PMC8586157; doi:10.1038/s41598-021-01137-1)
Supplement: Supplementary file 1 — Supplementary Information. [file 41598_2021_1137_MOESM1_ESM.docx]

**Supplementary Information**

**Optimization of an ammonia assay based on transmembrane pH-gradient polymersomes**

Anastasia Spyrogianni^‡^, Charlotte Gourmel^‡^, Leopold Hofmann, Jessica Marbach, Jean-Christophe Leroux^*^

Institute of Pharmaceutical Sciences, Department of Chemistry and Applied Biosciences, ETH Zurich, Vladimir-Prelog-Weg 3, CH-8093 Zurich, Switzerland

^‡^C. Gourmel and A. Spyrogianni contributed equally to this work

*Author to whom correspondence should be addressed: [jleroux@ethz.ch](mailto:jleroux@ethz.ch)

# Supplementary Methods

## Synthesis of polyethylene glycol monomethyl ether 2-bromoisobutyrate (mPEG-Br)

mPEG (Sigma-Aldrich) with number-averaged molecular weight (M_n_) 2,000 (25 g, 12.5 mmol, 1 eq) was dried under vacuum for 2 h at 70 °C. After flushing the flask with Ar, dry tetrahydrofuran (THF, Fisher) (130 mL) and trimethylamine (8.71 mL, 62.5 mmol, 5 eq, Fisher) were added while cooling back to room temperature. α-bromo isobutyryl bromide (BIBB, Sigma-Aldrich) (7.60 mL, 62.5 mmol, 5 eq) was diluted in dry THF (20 mL) in a small vial, which was previously flushed with Ar, and added dropwise with a syringe over 15 min to the mPEG reaction mixture. After 22 h stirring at room temperature, the excess BIBB was hydrolyzed by addition of a small quantity of water over 30 min. The mixture was filtered under vacuum to remove the trimetylammonium salt and the filtrate concentrated under vacuum. The product was then mixed with a small quantity of dichloromethane (DCM, Fisher), washed with saturated solutions of NaHCO_3_ (Sigma-Aldrich) (x2) and NaCl (Sigma-Aldrich) (x2) and dried over anhydrous Mg_2_SO_4_. The product was filtered, concentrated under vacuum, and precipitated in freezer cold (-20 °C) hexane (Fisher) (x1) and diethyl ether (Fisher) (x3). Finally, the product was dried extensively under vacuum to isolate mPEG2000-Br as a white solid with a yield ≥ 95% and a mass recovery of 75–80%. ^1^H NMR (400 MHz, chloroform-d_1_) δ 4.30-4.33 (m, 2H), 3.65 (s, 190H), 3.37 (s, 3H), 1.93 (s, 6H).

## Synthesis of poly(styrene)-*b*-PEG (PS-*b*-PEG) diblock copolymers

In a typical experiment, mPEG2000-Br (4.00 g, 2 nmol, 1 eq), 4,4’-dinonyl-2,2’-dipyridyl (1.08 g, 2.64 nmol, 1.32 eq), and Cu(I)Br (430 mg, 3 nmol, 1.5 eq) were weighed in a 50 mL Schlenk flask equipped with a magnetic bar and sealed with a septum. The reagents were then placed under Ar via three vacuum-Ar cycles. Styrene (15 mL, 130 nmol, 65 eq) was degassed by Ar-bubbling for 10 min prior to its addition to the Schlenk flask and the mixture was further deoxygenated via 3 freeze-pump-thaw cycles. The flask was then filled with Ar, sealed, and placed in a preheated oil bath (115 °C) for 19 h. The product (PS-*b*-PEG) was subsequently dissolved in THF while cooling down and filtered over basic alumina to remove copper. THF was evaporated under vacuum and the residue was dissolved in a small amount of DCM. The product was precipitated in freezer cold (-20 °C) hexane (x1) and diethylether (x3) afterwards. Finally, the polymer was extensively dried under vacuum and obtained as a white to slightly yellow solid. The degree of polymerization was tuned by adjusting the monomer to initiator ratio. A conversion of 50–70% was obtained.

## Gel permeation chromatography (GPC)

For GPC analysis, polymer samples were dissolved in THF at 2 mg/mL and solutions were filtered through polytetrafluoroethylene (PTFE) 0.2 µm filters prior to injection. The analysis was performed on the GPCmax VE2001 system (Viscotek, TX, USA) equipped with two ViscoGEL columns (TSKmax GMH_HR_-M poly(styrene-co-divinylbenzene) mixed bed), using 35 °C and THF at 0.5 mL/min. The GPC was coupled to a refractive index detector (TDA 302, Viscotek). The system was calibrated with poly(methyl methacrylate) standards (2,500–89,300 g/mol, PSS Polymer Standards Service, Germany) in the same solvent.

## PoSo purification

### General procedure

For PoSo made for ammonia sensing, the outer medium was phosphate buffer (50 mM) for 6.5 < pH_out_ $\text{≤}$ 8.0 or borate buffer (50 mM) for pH_out_ > 8.0. For PoSo made for optical property studies, the outer medium was CA buffer (5.5 mM) for pH_out_ $\text{≤}$ 6.5, phosphate buffer (50 mM) for 6.5 < pH_out_ $\text{≤}$ 8.0, or borate buffer (50 mM) for pH_out_ > 8.0. The osmolality of all buffer solutions was adjusted to 300 mOsm/kg using NaCl.

The PD-10 columns were first washed with 25 mL outer medium. When larger batches were needed, the eluates of multiple columns were pooled to obtain the final PoSo batch to be characterized and used for ammonia sensing.

### PoSo-HPTS

During purification, 1 mL PoSo-HPTS suspension and 1.5 mL outer medium were added to the desalting column and the flow through was discarded, after which 1.7 mL outer medium was added and the eluate containing the PoSo-HPTS was collected.

### PoSo-hCy

For PoSo-hCy, a pre-purification step with Lewatit^®^ VP OC 1064 MD PH adsorbent beads (Lanxess, Germany) was included (~200 mg beads/mL suspension) by vortexing the unpurified PoSo-hCy with the resin for 10 min. Thanks to the pre-purification, PD-10 column sample loading could be increased while keeping a good performance: *i.e.* 2.5 mL PoSo-hCy suspension and 0.5 mL outer medium were loaded, the flow through was discarded and the elution was performed with 3 mL outer medium.

### PoSo-Lyso

During purification, 0.5 mL PoSo-Lyso suspension and 2 mL outer medium were added to the desalting column and the flow through was discarded, after which 1.4 mL outer medium was added and the eluate containing the PoSo-Lyso was collected.

### PoSo-SNARF

The purification procedure was identical to PoSo-HPTS.

## **Polymer quantification in PoSo suspensions**

For PoSo-HPTS, a stock solution (2 mg/mL) of the polymer batch used for PoSo-HPTS synthesis was prepared in a mixture of outer medium buffer (2 or 5 vol%) in *N,N*-dimethylformamide (DMF, Acros Organics, Thermo Fisher Scientific). The stock solution was then appropriately diluted with the buffer/DMF mixture to prepare a series of polymer calibrants (5 non-zero levels, 0.03–1 mg/mL), while aliquots of the PoSo-HPTS suspension were mixed with DMF to obtain a final suspension concentration of 2 or 5 vol%. Calibrants and samples were vortexed vigorously and centrifuged (18,000 x *g*, 4 °C, 10 min). The absorbance of the supernatants was measured at 270 nm in UV-star^®^ 96-well transparent microplates (Greiner Bio-One, Switzerland) (100 µL/well) using the Infinite^®^ M200 or M200 Pro plate reader (Tecan, Switzerland). For PoSo-hCy, a similar procedure was followed but instead of DMF alone a DMF/dimethyl sulfoxide (DMSO) solvent mixture at a 1:1 volume ratio was used to prepare the calibrants and PoSo samples. Furthermore, the calibration curve was extended to 2 mg/mL (8 non-zero calibrants) and the centrifugation time was reduced to 8 min, which was sufficient for sedimentation of the insoluble buffer salts in this buffer/solvent mixture.

## Dye quantification in PoSo suspensions

### PoSo-HPTS

For PoSo-HPTS, calibration standard solutions (8 non-zero levels, 1–50 µM) of HPTS in outer buffer were prepared from a 100 µM HPTS stock solution in the same buffer. Aliquots of each calibrant and the PoSo suspension were then diluted to 2 vol% in DMF, vortexed vigorously, and centrifuged (18,000 x *g*, 4 °C, 10 min). The fluorescence of the supernatants (100 µL/well) was measured (λ_exc_ = 370 nm, λ_em_ = 416 nm) in polypropylene 96-well black microplates (Greiner Bio-One) using the Infinite^®^ M200 or M200 Pro plate reader (Tecan).

### PoSo-hCy

For PoSo-hCy, a similar procedure to that of PoSo-HPTS was followed but with the following modifications: hCy calibrants (8 non-zero levels, 20–180 µM) were prepared from a 2 mM hCy stock solution in outer medium buffer, a DMF:DMSO mixture (1:1 by volume) was used instead of DMF alone, the centrifugation time was reduced to 8 min, and fluorescence was measured at λ_exc_ = 625 nm, λ_em_ = 735 nm.

### PoSo-Lyso

For PoSo-Lyso, calibration standard solutions (8 non-zero levels, 3–100 µM) of Lyso-pH in outer medium buffer were prepared from a 100 µM Lyso-pH stock solution in the same buffer. Aliquots of each calibrant and the PoSo suspension were then diluted to 5 vol% in DMF, vortexed vigorously, and centrifuged (18,000 x *g*, 4 °C, 10 min). The fluorescence of the supernatants was measured (λ_exc_ = 655 nm, λ_em_ = 755 nm).

### PoSo-SNARF

In the case of PoSo-SNARF, the nominal dye concentration was used as these PoSo could not be used for ammonia sensing. Notably, quantification in DMF is not possible as SNARF™-4F loses absorbance in this solvent.

# Supplementary Figures


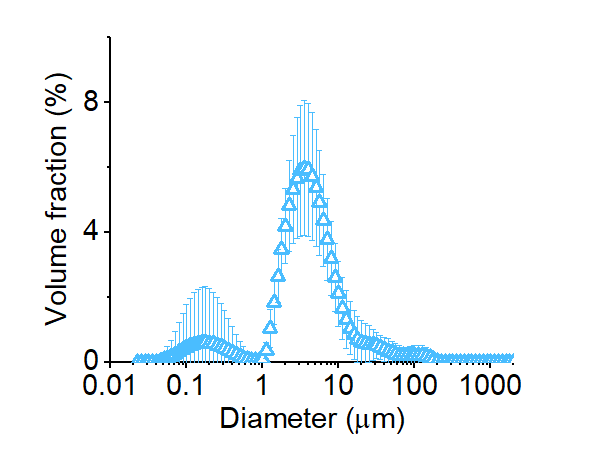


**Figure S1. PoSo characterization.** Representative size distributions by laser diffraction for PoSo-HPTS suspensions. Results are shown as mean ± SD (n=3) of replicate measurements.


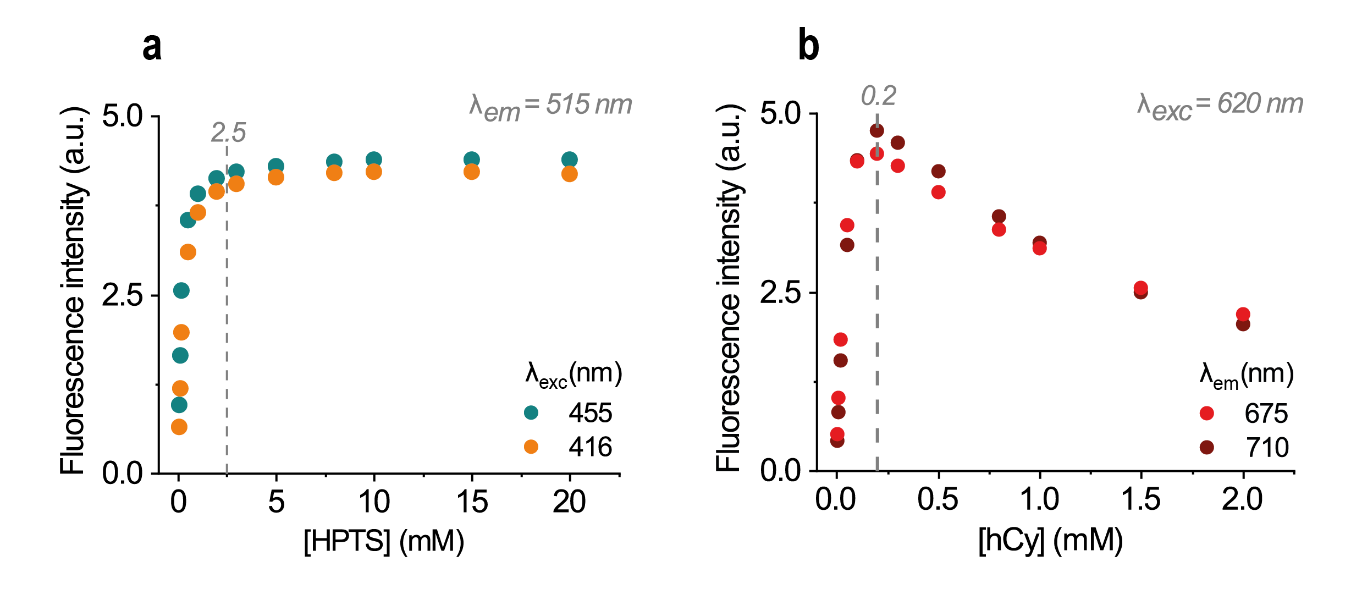


**Figure S2. Assessment of the self-quenching behavior of HPTS and hCy. a)** Fluorescence intensity (λ_exc_ 416 nm or 455 nm, λ_em_ = 515 nm) at increasing concentrations of HPTS. **b)** Fluorescence intensity (λ_exc_ = 620 nm, λ_em_ = 675 nm or 710 nm) at increasing concentration of hCy. While the fluorescence of HPTS reaches a plateau above 2.5 mM, hCy displays a self-quenching behaviour at a 10-fold lower concentration (0.2 mM). Samples were measured in PBS pH 7.4. An optimal gain setting was used.


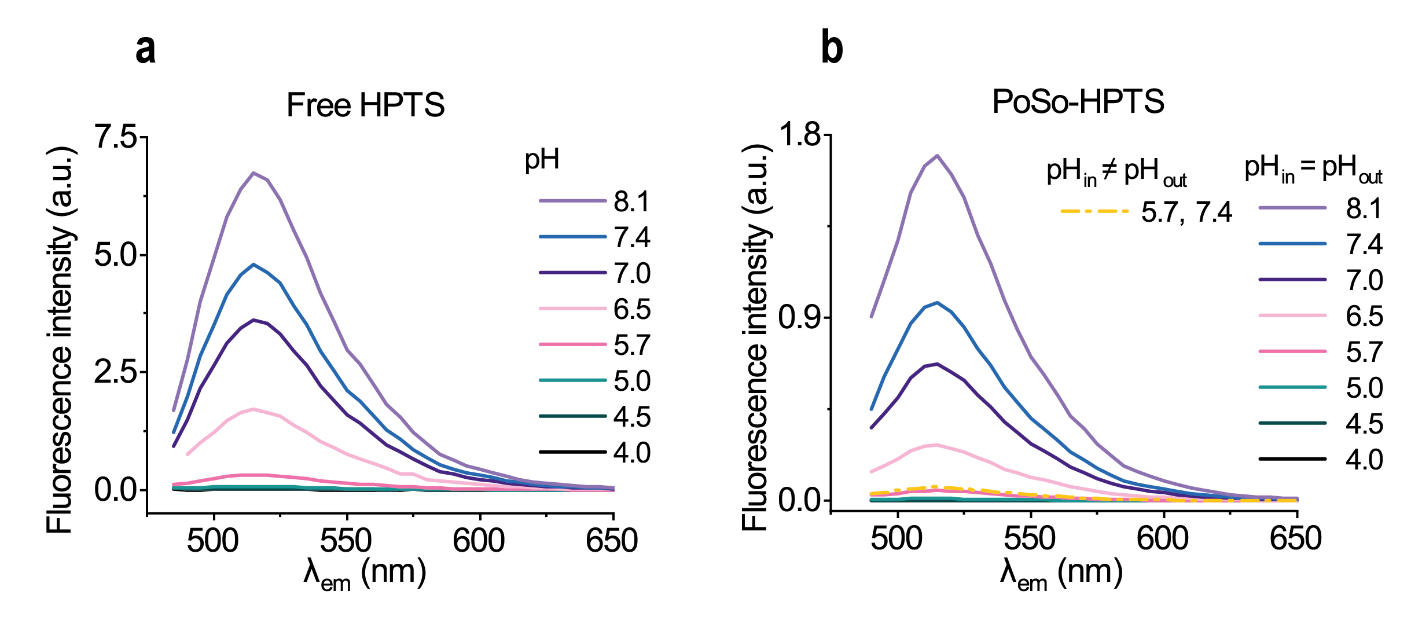


**Figure S3. Optical properties of free HPTS *vs.* PoSo-HPTS.** Fluorescence emission spectra (for λ_exc_ = 455 nm) of free HPTS (a) and PoSo-HPTS (b) with pH_in_ = pH_out_ (solid lines) and one sample with pH_in_ ≠ pH_out_ (dashed line) for comparison.

**
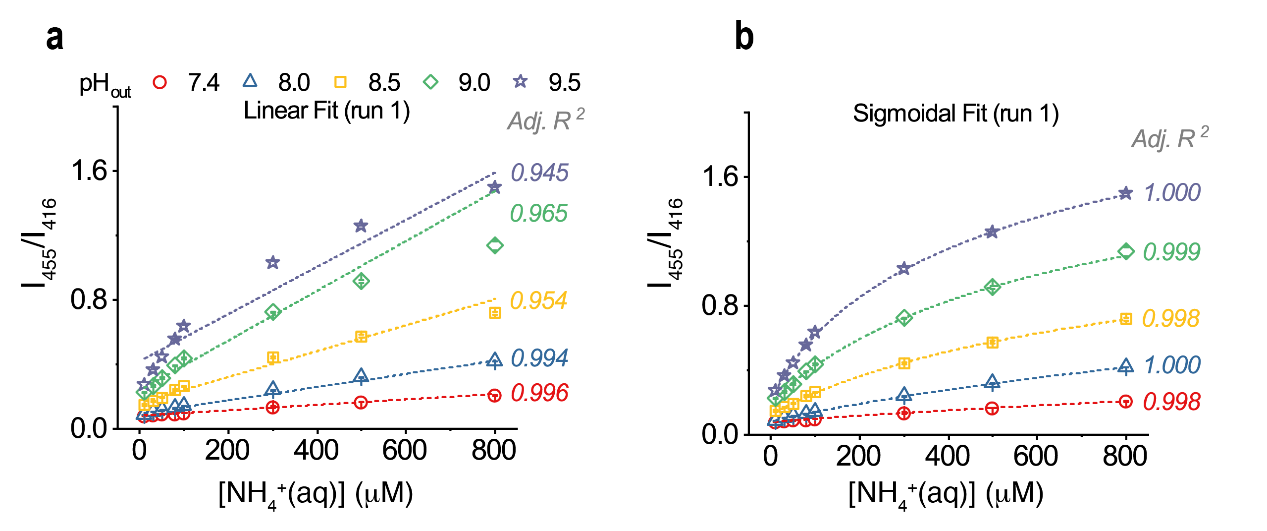
**

**Figure S4. PoSo-HPTS ammonia assay calibration curves with pH_in_ 5.7 and varying pH_out_.** **a-b)** Measured fluorescence intensity ratios I_455_/I_416_ (λ_em_ = 515 nm) (symbols) as a function of [NH_4_^+^_(aq)_] in calibrant solutions (10–800 μΜ) using PoSo-HPTS with pH_in_ 5.7 and various pH_out_. Calibration curves and coefficients of determination (Adj. R^2^) were obtained with either linear (a) or sigmoidal (b) regression. For each pH_out_, results are presented as mean ± SD (n=3) of replicate wells from one experimental run (run 1); error bars may be smaller than symbols. Conditions: [HPTS]_in_ = 1 mM, [HPTS]_assay_ = 0.4 µM, φ = 25 vol%.

*
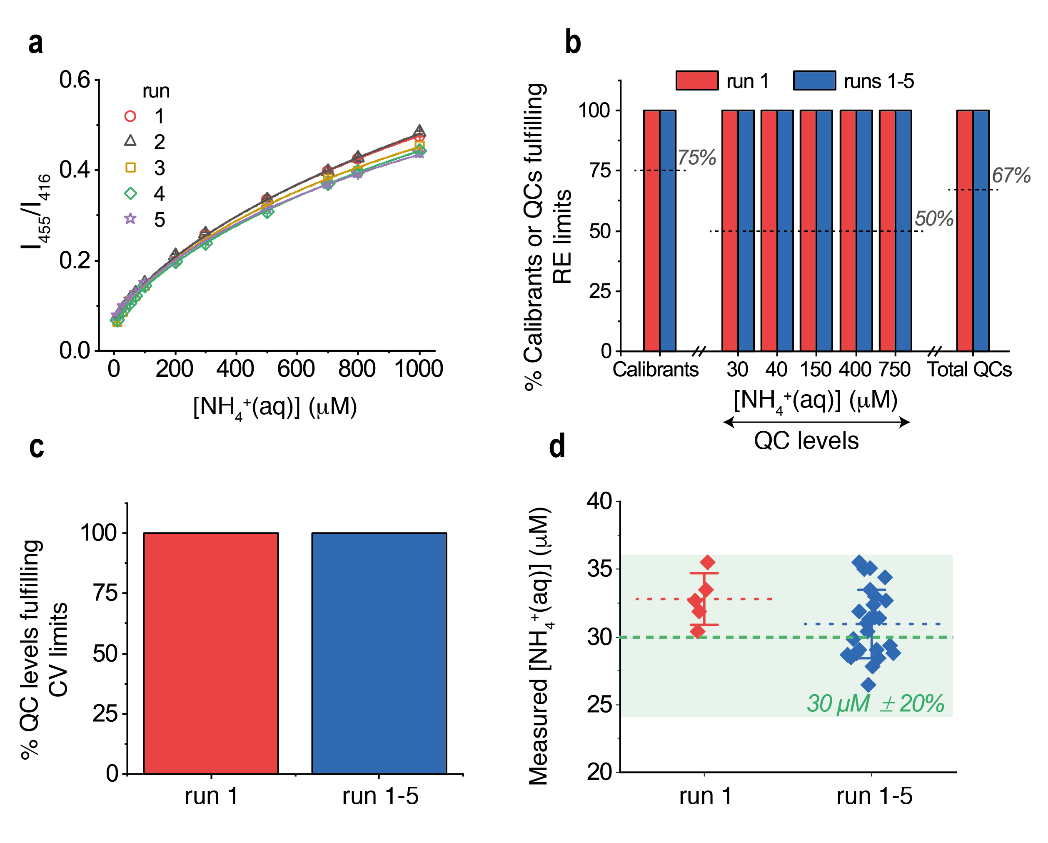
*

**Figure S5. PoSo-HPTS ammonia assay validation on 5 analytical runs and confirmation of LLOQ.** **a)** Ammonia sensing calibration curves obtained with sigmoidal regression fit of the measured fluorescence intensity ratio I_455_/I_416_ (λ_em_ = 515 nm) data plotted over [NH_4_^+^_(aq)_] in the calibrant solutions. Five independent analytical runs were performed over five consecutive days. Nine calibration levels within 30–800 µM NH_4_^+^_(aq)_ were used, while 10 and 1000 μM standards served as anchor points. Results correspond to mean ± SD (n=3) of replicate wells for each run; error bars may be smaller than symbols. Conditions: pH_in_ 6.0, pH_out_ 8.2, φ = 40 vol%, [HPTS]_in_= 1 mM, [HPTS]_assay_ = 0.4 µM. **b)** Fraction of calibration levels (n = 9), independently prepared QC replicates (n=5) per level, and total QCs (5 levels, n=5 replicates per level) with assay-determined [NH_4_^+^_(aq)_] having RE within ±15% (or ±20% for LLOQ) in run 1 (N=1) and runs 1–5 (N=5). Dotted lines indicate the minimum acceptable fractions in each category in accordance with EMA^24^ and FDA^23^. **c)** Fraction of QC levels with assay-determined [NH_4_^+^_(aq)_] having CV within ±15% (or ±20% for LLOQ) in run 1 and runs 1–5. **d)** Assay-determined [NH_4_^+^_(aq)_] for the LLOQ QC (30 µM) in run 1 (N=1, n=5) and runs 1–5 (N=5, n=5). Results are shown as exact measured values and mean ± SD (n=5) of independently prepared replicates. The nominal [NH_4_^+^_(aq)_] with ±20% RE are depicted as green dashed line and shaded area.


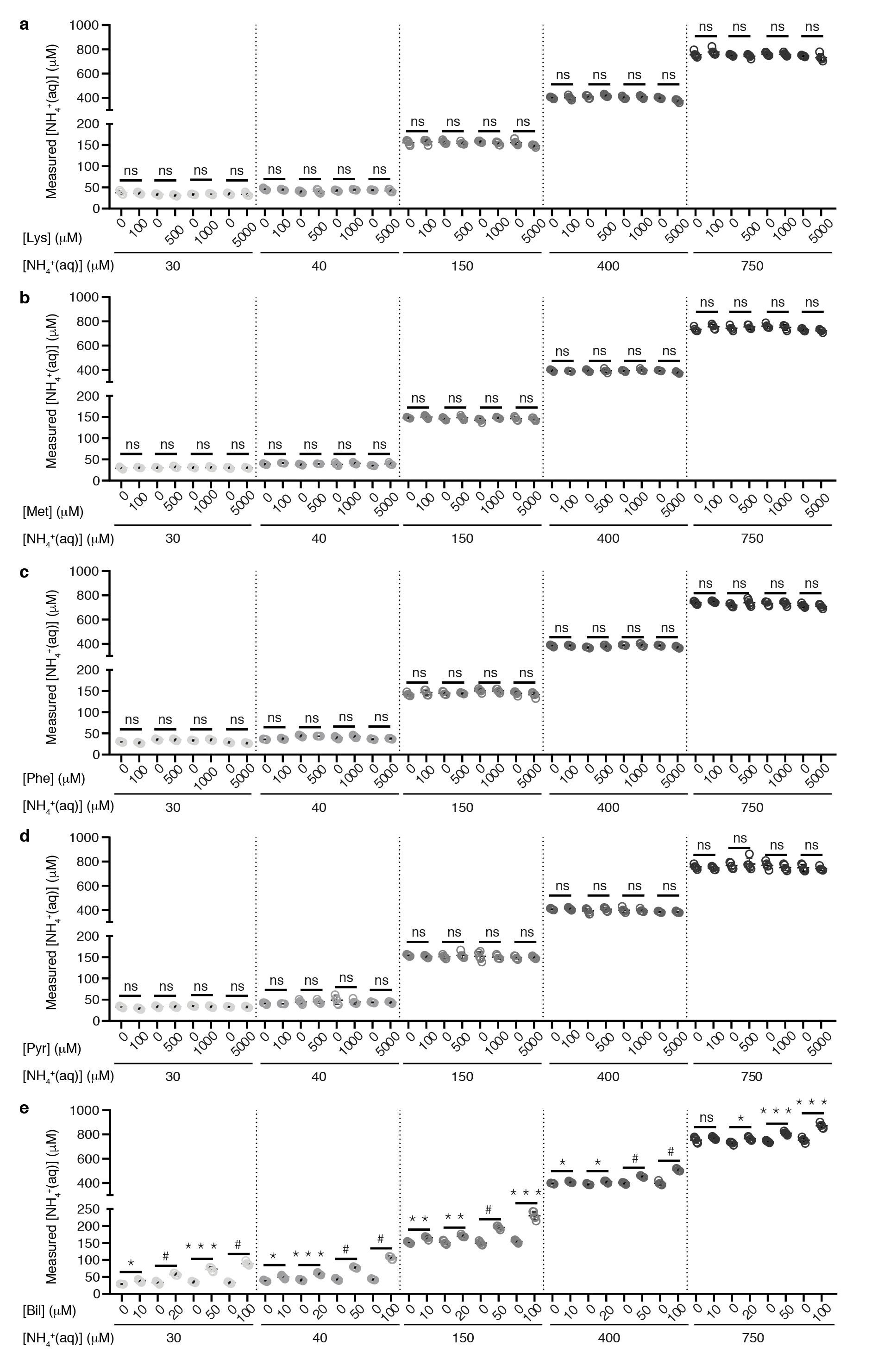


Figure S6. Analytical selectivity screening. Measured [NH_4_^+^_(aq)_] in the absence and presence of each interferent concentration for solutions containing 30, 40, 150, 400 or 750 μM NH_4_^+^_(aq)_ in PBS. The studied interferents were: Lys (a), Met (b), Phe (c), Pyr (d), and Bil (e). Results are shown as exact measured values and mean ± SD (n = 5 independently prepared QC replicates with or without interferent); error bars may be smaller than symbols. The additional data presented here for Lys, Pyr, and Bil as compared to Figure 4 are those for [NH_4_^+^_(aq)_] of 40 and 400 μM. Conditions: pH_in_ 6.0, pH_out_ 8.2, φ = 40 vol%, [HPTS]_in_= 1 mM, [HPTS]_assay_ = 0.4 µM. For each interferent, Brown-Forsythe and Welch ANOVA (parametric; unmatched) statistical analysis was performed with Holm-Sidak’s multiple comparison tests, where ns, *, **, *** and # correspond to p>0.05, p≤0.05, p≤0.01, p≤0.001 and and p≤0.0001, respectively.


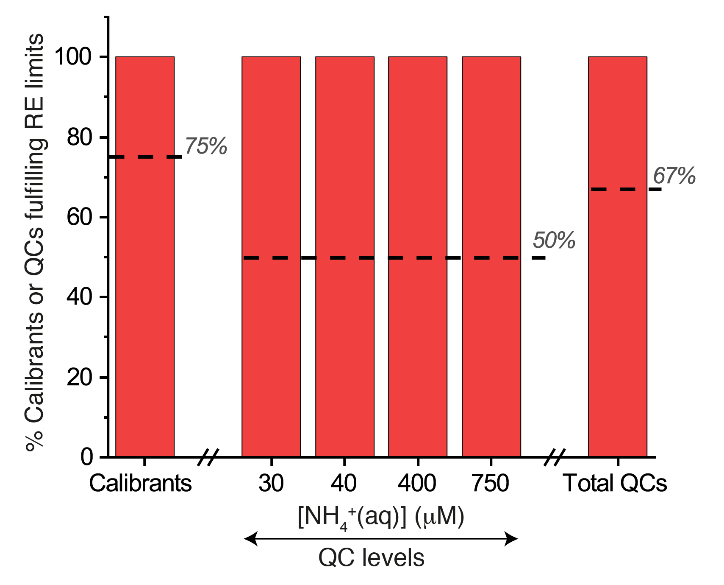


Figure S7. Ammonia assay performance in PBS using modified PoSo-HPTS. Fraction of calibration levels (10 levels, 30­–800 μM NH_4_^+^_(aq)_ in PBS), independently prepared QC replicates (n=4) per level, and total QCs (4 levels, n=4 replicates per level) with assay-determined [NH_4_^+^_(aq)_] having RE within ±15% (or ±20% for LLOQ). Dashed lines indicate the minimum acceptable fractions in each category in accordance with EMA^24^ and FDA^23^. Conditions: pH_in_ 6.0, pH_out_ 8.2, φ = 40 vol%, [HPTS]_in_ = 2.5 mM, [HPTS]_assay_ = 2.5 µM.


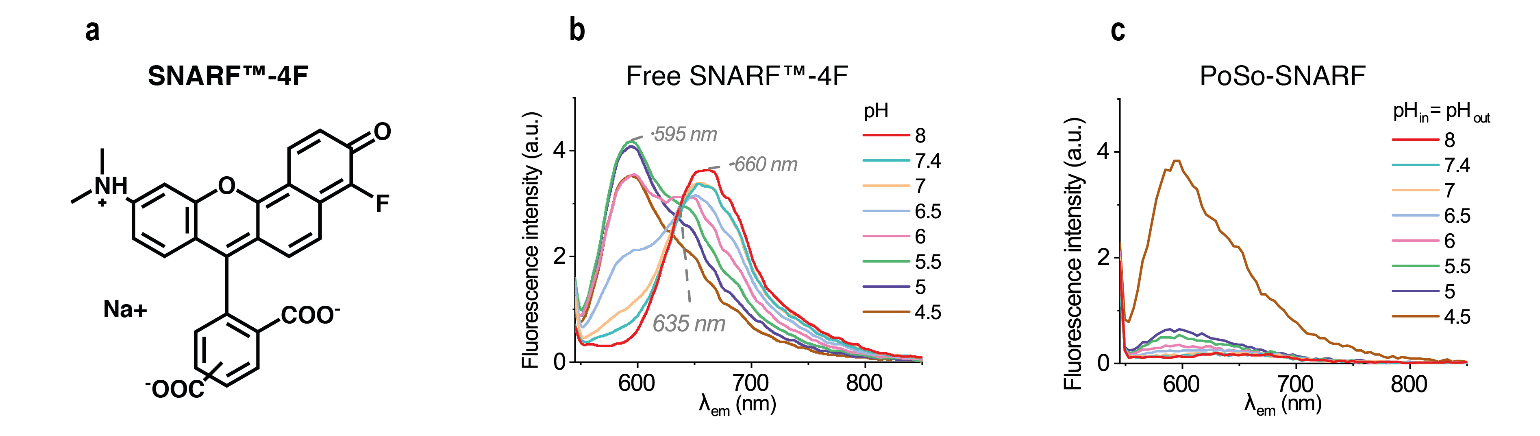


**Figure S8. Fluorescence properties of SNARF™-4F and PoSo-SNARF.** **a)** Structure of SNARF™-4F. **b-c)** Fluorescence emission spectra (λ_exc_ 515 nm) at various pH for free SNARF^TM^-4F (b) and for PoSo-SNARF with pH_in_ = pH_out_ (c).


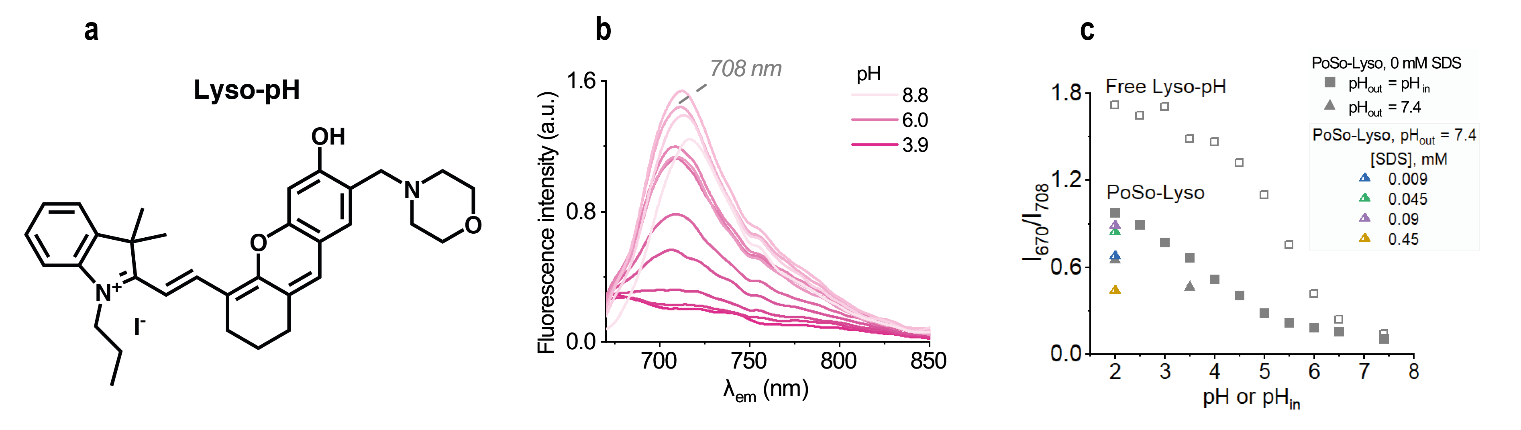


**Figure S9.** **Fluorescence properties of Lyso-pH and PoSo-Lyso. a)** Structure of Lyso-pH. **b)** Emission spectra (λ_exc_ = 635 nm) of free Lyso-pH in solution at different pH. **c)** Fluorescence intensity ratios I_670_/I_708_ *vs.* pH for free Lyso-pH (open squares) or *vs.* pH_in_ for PoSo-Lyso prepared with pH_in_ = pH_out_ (filled squares), PoSo-Lyso prepared with pH_out_ 7.4 (filled triangles), and PoSo-Lyso prepared with pH_in_ 2, pH_out_ 7.4, as well as incorporating different amounts of SDS (half-filled, colored triangles). An [SDS] of 0.09 mM was identified as the optimal concentration for minimizing potential interactions of the dye with the polymeric bilayer.


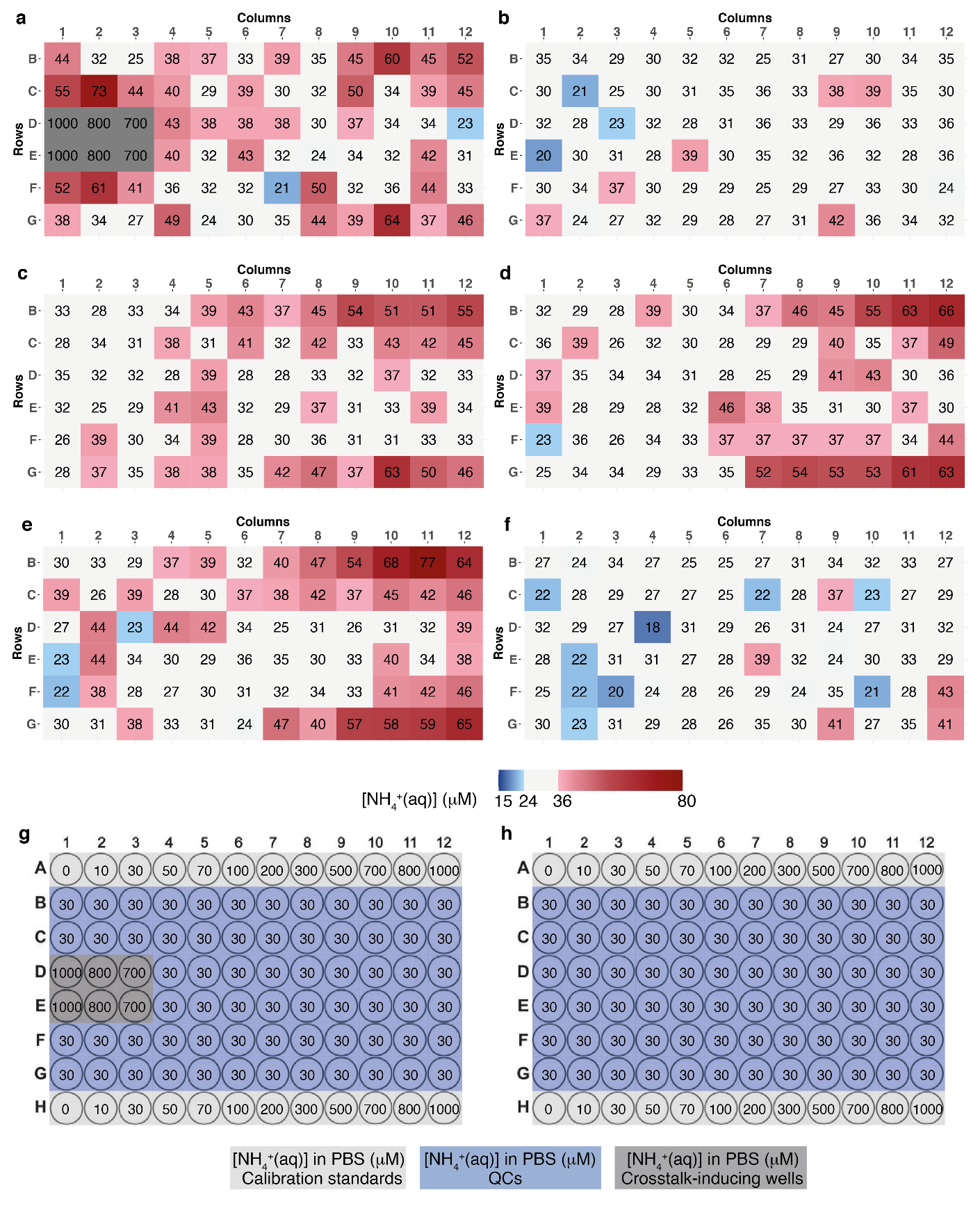


Figure S10. Ammonia assay well-to-well cross-talk phenomenon: ammonia quantification by PoSo-hCy in each well of a 96-well plate. Plate contains a 30 µM NH_4_^+^_(aq)_ QC sample in wells B1 to G12 and is represented as heatmaps and plate configuration. a) Back-calculated ammonia concentration using the PoSo-hCy assay in the following conditions: pH_in_ 6.5, pH_out_ 10, φ = 40 vol%, [hCy]_in_ = 250 μM, [Polymer]_assay_ = 1.3 mg/mL, 200 μL/well, incubation 10 min. The heatmap shows as white the wells where the measured ammonia concentration complies with the EMA^24^ and FDA^23^ criteria, blue for an underestimation below acceptable limits (±20% of nominal value), and red for an overestimation above these. The plate configuration, which included two calibration curves in row A and H, is shown in g). Wells D1–D3 and E1–E3 contain high values calibrants of 1000, 800 and 700 µM, inducing artifactually high ammonia measurements in neighboring wells, illustrating the phenomenon of ammonia well-to-well cross-talk. In fact, this is also observed in the wells surrounding the high level calibrants of the calibration curve. b-e) Back-calculated ammonia concentration using the PoSo-hCy assay in the same conditions as in (a) but with varying incubation time: 1.5 min (b), 10 min (c), 15 min (d), and 20 min (e), with the plate configuration shown in h). f) Back-calculated ammonia concentration using the PoSo-hCy assay in the following conditions: pH_in_ 6.5, pH_out_ 9.5, φ = 40 vol%, [hCy]_in_ = 250 μM, [Polymer]_assay_ = 1.3 mg/mL, incubation 5 min. These conditions were selected for ammonia measurements in human plasma. g) Microplate configuration and nominal ammonia concentration used in a). h) Microplate configuration and nominal ammonia concentration used in b-f). Calibrant and QC solutions were prepared in PBS pH 7.4.


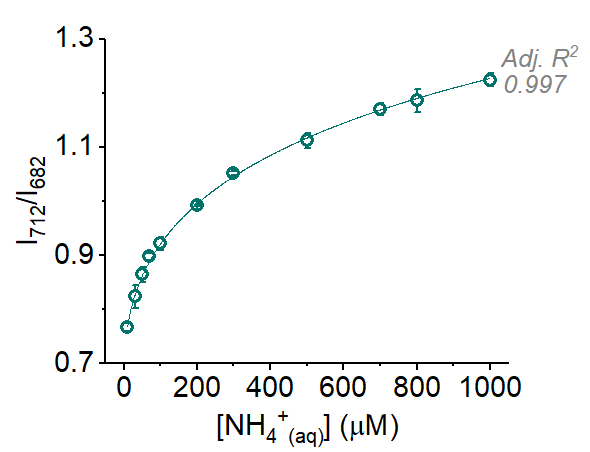


**Figure S11.** Representative ammonia sensing calibration curve obtained with sigmoidal regression fit of the measured fluorescence intensity ratios I_712_/I_682_ (λ_exc_ = 630 nm) data plotted over [NH_4_^+^_(aq)_] in PBS. Results correspond to mean ± SD (n=3) of replicate wells. Conditions: pH_in_ 6.5, pH_out_ 9.5, φ = 40 vol%, [hCy]_in_= 250 μM, C_pol,assay_ = 1.3 mg/mL, 200 μL/well.

# Supplementary Tables

Table S1. Mean [NH_4_^+^_(aq)_], accuracy (mean RE), and precision (CV) retrieved from: a) run 1 (N=1, n=5) or b) runs 1–3 (N=3, n=5), corresponding to the assays described in Figure 2.

| 1. **Within-run (run 1)** | | | | | | | | | | | | | | | | |
| --- | --- | --- | --- | --- | --- | --- | --- | --- | --- | --- | --- | --- | --- | --- | --- | --- |
| Nominal  [NH_4_^+^_(aq)_]  (µM) | Fit | Mean  [NH_4_^+^_(aq)_] (µM) | | | | | Mean RE (%) | | | | | CV (%) | | | | |
|  | *pHo* | *7.4* | *8.0* | *8.5* | *9.0* | *9.5* | *7.4* | *8.0* | *8.5* | *9.0* | *9.5* | *7.4* | *8.0* | *8.5* | *9.0* | *9.5* |
| 60 | Linear | 89 | 70 | 80 | 85 | 70 | 48 | 17 | 33 | 42 | 16 | 9 | 3 | 5 | 7 | 10 |
|  | Sigmoidal | 72 | 69 | 64 | 70 | 65 | 21 | 15 | 7 | 16 | 9 | 9 | 3 | 3 | 4 | 5 |
| 400 | Linear | 461 | 370 | 454 | 483 | 431 | 15 | -7 | 14 | 21 | 8 | 2 | 1 | 2 | 2 | 2 |
|  | Sigmoidal | 435 | 422 | 405 | 430 | 391 | 9 | 6 | 1 | 7 | -2 | 3 | 1 | 3 | 4 | 3 |
| 750 | Linear | 718 | 566 | 666 | 657 | 595 | -4 | -25 | -11 | -12 | -21 | 3 | 1 | 1 | 2 | 1 |
|  | Sigmoidal | 761 | 774 | 788 | 740 | 756 | 2 | 3 | 5 | -1 | 1 | 3 | 2 | 1 | 4 | 3 |

| 1. **Between-run (runs 1–3)** | | | | | | | | | | | | | | | | |
| --- | --- | --- | --- | --- | --- | --- | --- | --- | --- | --- | --- | --- | --- | --- | --- | --- |
| Nominal  [NH_4_^+^_(aq)_]  (µM) | Fit | Mean  [NH_4_^+^_(aq)_] (µM) | | | | | Mean RE (%) | | | | | CV (%) | | | | |
|  | *pHo* | *7.4* | *8.0* | *8.5* | *9.0* | *9.5* | *7.4* | *8.0* | *8.5* | *9.0* | *9.5* | *7.4* | *8.0* | *8.5* | *9.0* | *9.5* |
| 60 | Linear | 91 | 76 | 65 | 76 | 59 | 52 | 27 | 9 | 26 | -2 | 16 | 13 | 17 | 36 | 43 |
|  | Sigmoidal | 84 | 69 | 65 | 69 | 67 | 40 | 16 | 9 | 14 | 11 | 17 | 7 | 4 | 4 | 5 |
| 400 | Linear | 459 | 425 | 459 | 491 | 498 | 15 | 6 | 15 | 23 | 25 | 3 | 10 | 3 | 9 | 10 |
|  | Sigmoidal | 434 | 429 | 412 | 416 | 399 | 8 | 7 | 3 | 4 | -0.3 | 4 | 2 | 3 | 4 | 3 |
| 750 | Linear | 733 | 649 | 678 | 689 | 698 | -2 | -13 | -10 | -8 | -7 | 3 | 9 | 2 | 8 | 11 |
|  | Sigmoidal | 762 | 764 | 770 | 749 | 749 | 2 | 2 | 3 | -0.2 | -0.2 | 3 | 2 | 3 | 4 | 4 |

**Table S2.** Mean [NH_4_^+^_(aq)_], accuracy (mean RE), and precision (CV) retrieved from: **a)** run 1 (N=1, n=5) or **b)** runs 1–3 (N=3, n=5), corresponding to the assays described in **Figure 3**.

| 1. **Within-run (run 1)** | | | | | | | | | | |
| --- | --- | --- | --- | --- | --- | --- | --- | --- | --- | --- |
| Nominal  [NH_4_^+^_(aq)_] (µM) | φ (vol%) | Mean  [NH_4_^+^_(aq)_] (µM) | | | Mean RE (%) | | | CV (%) | | |
|  | *pHo* | *8.0* | *8.2* | *8.5* | *8.0* | *8.2* | *8.5* | *8.0* | *8.2* | *8.5* |
| 45 | 10 | 62 | 59 | 56 | 37 | 31 | 24 | 16 | 8 | 8 |
|  | 25 | 56 | 38 | 50 | 25 | -15 | 12 | 13 | 8 | 4 |
|  | 40 | 48 | 50 | 49 | 7 | 11 | 8 | 5 | 3 | 6 |
| 400 | 10 | 413 | 408 | 390 | 3 | 2 | -3 | 5 | 5 | 5 |
|  | 25 | 416 | 371 | 395 | 4 | -7 | -1 | 2 | 2 | 3 |
|  | 40 | 376 | 390 | 402 | -6 | -3 | 0.4 | 2 | 5 | 3 |
| 750 | 10 | NA | 669 | 757 | NA | -11 | 1 | NA | 7 | 4 |
|  | 25 | 770 | 723 | 745 | 3 | -4 | -1 | 2 | 2 | 0.1 |
|  | 40 | 756 | 751 | 779 | 1 | 0.2 | 4 | 3 | 1 | NA |

| 1. **Between-run (runs 1–3)** | | | | | | | | | | |
| --- | --- | --- | --- | --- | --- | --- | --- | --- | --- | --- |
| Nominal  [NH_4_^+^_(aq)_] (µM) | φ (vol%) | Mean  [NH_4_^+^_(aq)_] (µM) | | | Mean RE (%) | | | CV (%) | | |
|  | *pHo* | *8.0* | *8.2* | *8.5* | *8.0* | *8.2* | *8.5* | *8.0* | *8.2* | *8.5* |
| 45 | 10 | 59 | 59 | 56 | 31 | 32 | 24 | 26 | 11 | 11 |
|  | 25 | 52 | 49 | 53 | 15 | 8 | 17 | 12 | 18 | 8 |
|  | 40 | 50 | 51 | 49 | 12 | 13 | 10 | 10 | 4 | 5 |
| 400 | 10 | 392 | 404 | 397 | -2 | 1 | -1 | 6 | 4 | 4 |
|  | 25 | 417 | 399 | 406 | 4 | -0.2 | 2 | 3 | 6 | 4 |
|  | 40 | 394 | 396 | 398 | -1 | -1 | -0.4 | 4 | 4 | 3 |
| 750 | 10 | 772 | 730 | 760 | 3 | -3 | 1 | 2 | 9 | 3 |
|  | 25 | 778 | 749 | 763 | 4 | -0.2 | 2 | 2 | 4 | 2 |
|  | 40 | 752 | 749 | 784 | 0.3 | -0.2 | 5 | 2 | 3 | 1 |

**Table S3.** RE on the mean [NH_4_^+^_(aq)_] of unspiked and ammonia-spiked healthy human plasma samples measured by the PoSo-HPTS ammonia assay (with either calibration curve) compared to the POC device in **Figure 6b**.

| **Spiked [NH_4_^+^_(aq)_] (μM)** | **RE vs. POC device (%)** | |
| --- | --- | --- |
|  | **PoSo-HPTS**  **(calibration curve in PBS)** | **PoSo-HPTS**  **(calibration curve in plasma)** |
| Unspiked | -26 | -26 |
| 0 | -23 | -23 |
| 50 | -18 | -13 |
| 60 | -22 | -17 |
| 80 | -19 | -11 |
| 120 | -19 | -8 |

**Table S4.** RE on the mean [NH_4_^+^_(aq)_] of unspiked and ammonia-spiked healthy human plasma samples measured by the PoSo-hCy ammonia assay compared to the POC device in **Figure 8b**.

| **Spiked [NH_4_^+^_(aq)_] (μM)** | **RE vs. POC device (%)** | |
| --- | --- | --- |
|  | **run 1** | **run 2** |
| Unspiked | 6 | -15 |
| 50 | 4 | -25 |
| 60 | -3 | -5 |
| 80 | 0.1 | 14 |
| 120 | 9 | -3 |
